# Investigation of reactive astrogliosis effect on post-stroke cognitive impairment

**DOI:** 10.1186/s12974-020-01985-0

**Published:** 2020-10-17

**Authors:** Kuo-Lun Huang, Ing-Tsung Hsiao, Meng-Yang Ho, Jung-Lung Hsu, Yeu-Jhy Chang, Ting-Yu Chang, Chi-Hung Liu, Chien-Hung Chang, Yi-Ming Wu, Kuan-Yi Wu, Shiaw-Pyng Wey, Tzu-Chen Yen, Nobuyuki Okamura, Tsong-Hai Lee, Kun-Ju Lin

**Affiliations:** 1 Department of Neurology, Linkou Chang Gung Memorial Hospital, and College of Medicine, Chang Gung University, No. 5, Fuxing St., Guishan, Taoyuan, Taiwan; 2 Department of Nuclear Medicine and Molecular Imaging Center, Linkou Chang Gung Memorial Hospital, No. 5, Fuxing St., Guishan, Taoyuan, Taiwan; 3 Healthy Aging Research Center and Department of Medical Imaging and Radiological Sciences, College of Medicine, Chang Gung University, Taoyuan, Taiwan; 4 Graduate Institute of Behavioral Sciences, Chang Gung University, Taoyuan, Taiwan; 5 Department of Neurology, New Taipei Municipal TuCheng Hospital, Chang Gung Memorial Hospital, Chang Gung University, New Taipei City, Taiwan; 6 Taipei Medical University, College of Humanities and Social Sciences, Graduate Institute of Humanities in Medicine and Research Center for Brain and Consciousness, Shuang Ho Hospital, Taipei, Taiwan; 7 Department of Radiology, Chang Gung Memorial Hospital, Taoyuan, Taiwan; 8 Department of Psychiatry, Chang Gung Memorial Hospital, Taoyuan, Taiwan; 9 Division of Neuro-imaging, Institute of Development, Aging and Cancer, Tohoku University, Sendai, Japan; 10 Division of Pharmacology, Faculty of Medicine, Tohoku Medical and Pharmaceutical University, Sendai, Japan

**Keywords:** Post-stroke cognitive impairment, Reactive astrogliosis, PET, Stroke

## Abstract

**Background:**

The aim of this study is to investigate the associations between post-stroke cognitive impairment (PSCI) severity and reactive astrogliosis (RA) extent on normalized ^18^F-THK-5351 positron-emission tomography (PET) imaging in amyloid-negative patients with first-ever stroke**.**

**Methods:**

We prospectively enrolled 63 amyloid-negative patients with first-ever stroke. Neurocognitive evaluation, MRI, ^18^F-THK-5351, and ^18^F-florbetapir PET were performed around 3 months after stroke. The ^18^F-THK-5351 uptake intensity was normalized using a signal distribution template to obtain the Z-SUM scores as the RA extent in the whole brain and cerebral hemisphere ipsilateral to stroke lesion. We evaluated stroke volume, leukoaraiosis, and brain atrophy on MRI. We used a comprehensive neurocognitive battery to obtain composite cognitive scores, and defined PSCI as a general cognitive function score < − 1. We analyzed the influence of Z-SUM scores on PSCI severity after adjusting for demographic, vascular, and neurodegenerative variables.

**Results:**

Twenty-five of 63 stroke patients had PSCI. Patients with PSCI had older age, lower education, and more severe cortical atrophy and total Z-SUM scores. Total Z-SUM scores were significantly associated with general cognitive and executive functions at multiple regression models. Path analyses showed that stroke can exert cognitive influence directly by stroke itself as well as indirectly through RA, including total and ipsilateral Z-SUM scores, in patients with either right or left hemisphere stroke.

**Conclusion:**

The patterns and intensity of ^18^F-THK-5351 uptake in amyloid-negative patients with first-ever stroke were associated with PSCI manifestations, which suggests that RA presents a modulating effect in PSCI development.

## Introduction

Post-stroke dementia (PSD) and post-stroke cognitive impairment (PSCI) affect up to one-third of stroke survivors and mostly occur within the first 6 months [1, 2]. PSD is not a single disease entity; rather, it describes an unspecified dementia syndrome that occurs after stroke [3]. Epidemiological studies have reported PSD etiologies to be attributed to Alzheimer’s disease (AD) or co-occurring AD and vascular dementia in 29% to 61% of patients with PSD [4]. According to the double-hit theory on PSCI development, patients with stroke have different post-stroke cognitive trajectories depending on their amyloid plaque burden and neuroinflammation severity [5]. Functional and molecular imaging has facilitated the understanding of the complex interactions between neurodegeneration and vascular injury in post-stroke cognitive presentations [6]. On the other hand, stroke, like other CNS injury, will induce a cascade of neuroinflammatory responses, and neuroinflammatory fluid biomarkers, such as C-reactive protein, IL-8 and IL-12, have been identified to be associated with PSCI occurrence [7, 8]. However, there is limited human neuroimaging evidence on the relationships among stroke lesions, neuroinflammatory severity, and PSCI [5, 9].

^18^F-THK-5351 is a radiotracer designed for in vivo tau protein detection in patients with AD [10]. In addition to tau protein binding, ^18^F-THK-5351 has been reported to have an affinity for monoamine oxidase-B (MAO-B), which might explain the off-target ^18^F-THK-5351 binding in the striatum, thalamus, and brainstem [11, 12]. Furthermore, MAO-B is largely expressed in astrocytes during the neuroinflammatory phenomenon of reactive astrogliosis (RA) [13]. Previous studies have reported increased ^18^F-THK-5351 uptake around stroke lesions, which might provide information regarding astrocyte-related neuroinflammatory changes [14, 15]. However, stroke-induced ^18^F-THK-5351 uptake signals might overlap with background signals, mainly in striatum and thalamus, thereby hindering the quantification of stroke-induced neuroinflammation on ^18^F-THK-5351 positron-emission tomography (PET) imaging.

Firstly, we aimed to establish a signal distribution template of ^18^F-THK-5351 PET imaging using healthy subjects, and subsequently apply the template in patients with stroke to diminish subcortical background signals through statistical transformation. Thereby, stroke-induced ^18^F-THK-5351 uptake could be quantified as a neuroinflammatory imaging biomarker of RA. Secondly, we aimed to explore the correlations between PSCI and neuroinflammation severity on ^18^F-THK-5351 PET imaging in amyloid-negative patients with first-ever stroke.

## Materials and methods

### Participants

We conducted a prospective, cross-sectional study to screen 72 patients with recent first-ever stroke (around 3 months after onset, median 87 days with interquartile range from 75 to 100 days) from the Department of Neurology and Stroke Center at Linkou Chang Gung Memorial Hospital, Taiwan, as previously described [15]. These stroke patients fulfilled the inclusion criteria: (1) a diagnosis of ischemic or hemorrhagic stroke confirmed on brain computed tomography (CT) or magnetic resonance imaging (MRI) at stroke onset; (2) no history of old stroke, dementia, tauopathy diseases, substantial traumatic brain injury, or epilepsy before the index stroke; and (3) the Informant Questionnaire on Cognitive Decline (IQCODE) mean score < 3.4 obtained within 1 week after stroke onset [16]. Nine of these patients were further excluded due to (1) failure to receive ^18^F-THK-5351 (*n* = 2) and ^18^F-florbetapir (*n* = 3) PET scanning, (2) amyloid-positivity on ^18^F-florbetapir PET scanning (*n* = 2), (3) recurrent stroke occurring between the index stroke and the study screening procedure (*n* = 2), and (4) persistent moderate to severe dysphasia, which was defined as a score of > 1 point in the language score of the National Institutes of Health Stroke Scale (NIHSS) [17]. Finally, 63 amyloid-negative patients with recent first-ever stroke were recruited after panel evaluation by neurologists, neuropsychologists, neuroradiologists, and experts in nuclear medicine. There was no significant difference in age, education, stroke volume, and the intervals from stroke onset to screening procedure between patients recruited and excluded.

The study protocol and procedure for obtaining informed consent were complied with the Helsinki Declaration, and were approved by the institutional review board of Chang Gung Memorial Hospital (IRB No. 103-7584A and 201601675A0) with the clinical trials registered to Taiwan Food and Drug Administration (1040025953 and 1066015148) and Center of Drug Evaluation (104IND06124 and 106IND03071). All participants provided written informed consent.

### Neurocognitive and functional evaluation

We employed a battery of neurocognitive tests, which had been used in previous studies [15, 18, 19], to assess a range of cognitive domains around 3 months after stroke onset. The neurocognitive tests in each cognitive domain were summarized in the Sup. Table 1. Of note, the IQCODE test was done twice in the current study; the first test was done within 1 week after stroke onset for pre-stroke cognitive state screening as one of the inclusion criteria, and the second test was done around 3 months after stroke onset for PSCI severity evaluation [20]. Depressive and anxiety symptoms were assessed by the Neuropsychiatric Inventory (NPI) items 4 and 5, respectively. The sequence of tests administration was identical for each individual participant to minimize any possible interference effect between testing. The assessment of disability states and cognitive domains mainly consisted of memory, language, executive, and visuospatial functions. Each raw test score was transformed to a *z* score based on its corresponding normative data. We derived the composite scores for the four cognitive domains (memory, visuospatial, executive, and language functions) by averaging the *z* scores of the relevant tests. We calculated the overall composite score for general cognitive function by averaging the *z* scores of all tests that contributed to the four cognitive domains. PSCI was defined as the overall composite score for general cognitive function < − 1 [21, 22].

### Imaging evaluation

#### Stroke volume evaluation

Brain CT and MRI were performed at stroke onset to assess acute stroke lesions. The MRI scanning protocol included fluid-attenuated inversion recovery (FLAIR), diffusion-weighted imaging (DWI), and T1-weighted (T1W) sequences. The stroke volume was delineated on the DWI maps for ischemic stroke and on the CT images for hemorrhagic stroke using the PMOD software (version 3.7; PMOD Technologies Ltd., Zurich, Switzerland). We normalized stroke lesion volume according to the head size, which was measured using the Freesurfer software (version 6.0.0).

#### Brain atrophy, leukoaraiosis, and vascular burden evaluation

Brain atrophy and leukoaraiosis were evaluated based on the follow-up brain MRI scans performed around 3 months after stroke onset. Axial three-dimensional T1W-MPRAGE (Magnetization Prepared RApid Gradient Echo), susceptibility-weighted imaging (SWI), and FLAIR sequences were acquired on a Siemens 3T MRI system as previously described [15].

We measured the cortical thickness on the T1W-MPRAGE images using the Freesurfer software [23]. We evaluated hippocampal atrophy using the Schelten medial temporal lobe atrophy (MTA) score [24, 25]. We measured leukoaraiosis severity on the FLAIR sequence over the periventricular and deep white matter areas for each cerebral hemisphere by the Fazekas scale [26]. Periventricular leukoaraiosis (PVL) was scored as follows: 0 = absence, 1 = caps or pencil-thin lining, 2 = smooth halo, or 3 = irregular leukoaraiosis extending into the deep white matter. Further, deep white matter leukoaraiosis (DWML) was scored as follows: 0 = absence, 1 = punctuate foci, 2 = beginning confluence of foci, or 3 = large confluent areas.

Other imaging biomarkers for small vessel disease were also rated by a senior neuroradiologist. Lobar and deep cerebral microbleeds (< 10 mm in diameter) were evaluated on SWI [27]. Presence of lacunes (3–15 mm in diameter) and cerebral microbleeds were defined as the presence of one or more lacunes or any cerebral microbleed [28]. Presence of enlarged perivascular space (< 3 mm in diameter) was counted if there was moderate to severe (grade 2–4) perivascular space in the basal ganglia [28, 29].

#### PET image acquisition and preprocessing procedures

Both ^18^F-florbetapir and ^18^F-THK-5351 positron-emission tomography (PET) scans were performed separately using Biograph mMR PET/magnetic resonance (MR) and mCT PET/computed tomography (CT) scanners (Siemens Medical Solutions, Malvern, PA, USA) about 3 months after stroke onset; further, the two scans were conducted at least 48 h apart to avoid signal interference. A 10-min PET scan of ^18^F-florbetapir was acquired at 50 min post-injection of 384 ± 13 MBq while a 10-min acquisition of ^18^F-THK-5351 was performed at 50 min post-injection of 379 ± 13 MBq. ^18^F-florbetapir PET images were reconstructed using point-spread function reconstruction with 2 iterations and 21 subsets, as well as MR-based attenuation correction and scatter and random corrections. Further, ^18^F-THK-5351 PET images were reconstructed using a 3-D ordered subsets-expectation maximization reconstruction algorithm (4 iterations, 24 subsets; Gaussian filter with 2 mm full width at half maximum, zoom 3) with CT-based attenuation correction, as well as scatter and random corrections. The final reconstructed images were of 344 × 344 × 127 matrix size (0.834 × 834 × 1.2 mm voxel size) for ^18^F-florbetapir and 400 × 400 × 148 matrix size (0.68 × 0.68 × 1.5 mm voxel size) for ^18^F-THK-5351.

PET data were motion-corrected, and then spatially normalized into MNI space using MR-based spatial normalization. Image processing was performed using PMOD software (version 3.7; PMOD Technologies Ltd, Zurich, Switzerland) by previously reported protocols [30]. Then, the standardized uptake value ratio (SUVR) image was calculated by using the cerebellar grey matter as the reference region. Amyloid plaque positivity was visually evaluated on ^18^F-florbetapir PET images [31].

#### Transformation of ^18^F-THK-5351 images to Z-Score map

We applied Z-score analysis to diminish the background signal from the subcortical regions resulting from the intrinsic signal distribution of ^18^F-THK-5351 images. To calculate the ^18^F-THK-5351 Z-score image for each subject, a dataset of ^18^F-THK-5351 PET SUVR images were first obtained from 22 age-matched healthy subjects to establish the ^18^F-THK-5351 signal distribution template as previously described [32–34]. These healthy subjects had (1) age > 55 years; (2) Mini-Mental State Examination (MMSE) score ≥ 26 points; (3) no history of stroke, cognitive impairment, parkinsonism, or other neurodegenerative diseases; (4) not taking anti-inflammatory medication during the imaging period; and (5) negativity for amyloid plaque on ^18^F-florbetapir PET scanning. These healthy subjects were 65.1 (6.1) years old, with MMSE score of 28.0 (1.1) points. Ten of them were female.

**Fig. 1:**
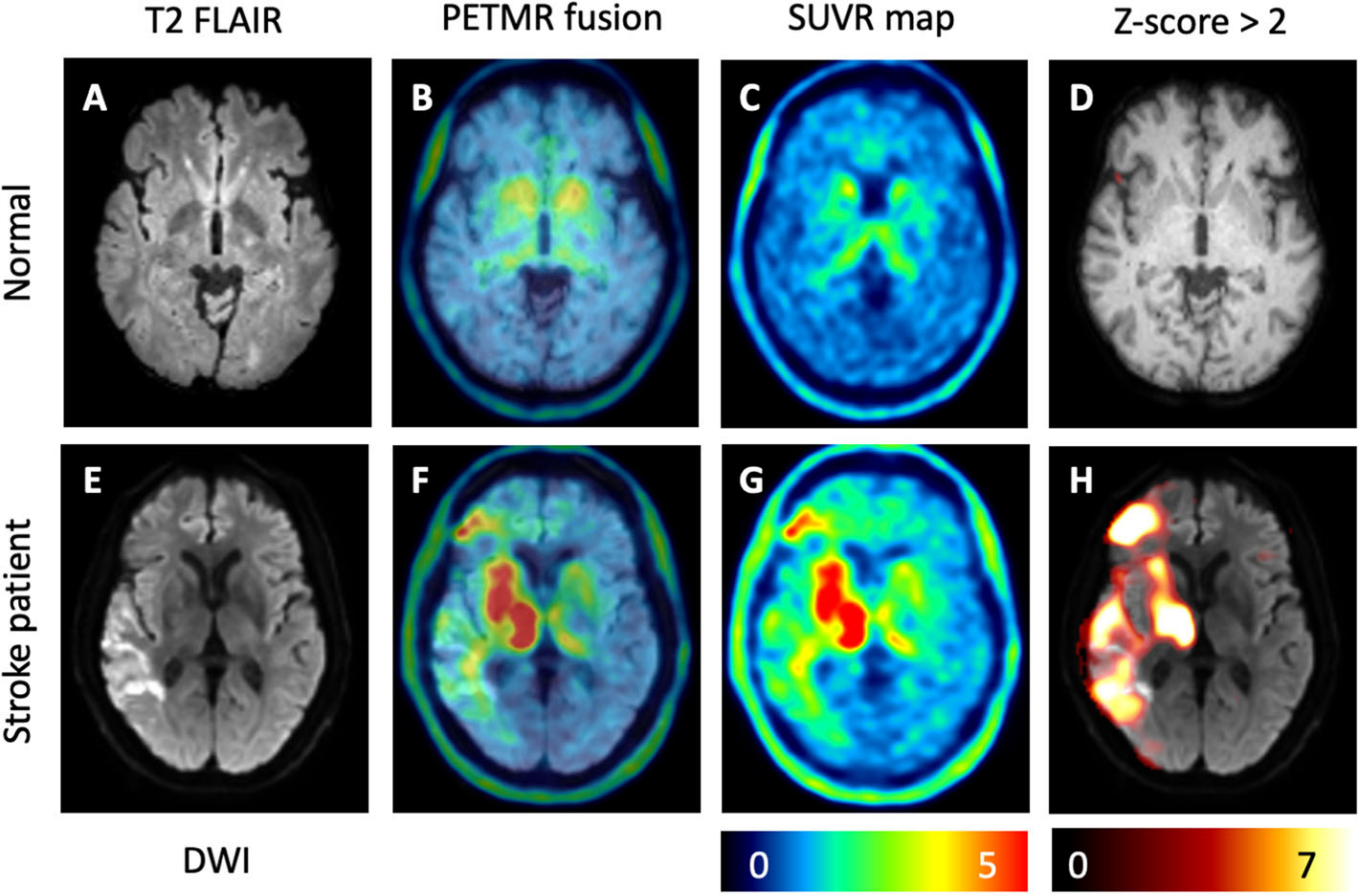
Transforming the ^18^F-THK-5351 SUVR maps to ^18^F-THK-5351 Z-score maps. Regarding a representative healthy subject (**a**), off-target binding of ^18^F-THK-5351 to the basal ganglia and thalamus was noted on the SUVR maps (**b**, **c**). After transforming SUVR maps to Z-score maps, the signals in the basal ganglia and thalamus were robustly diminished (**d**). Regarding a patient with right hemisphere ischemic stroke (**e**), ^18^F-THK-5351 uptake signals were increased around the stroke lesion; further, they were asymmetrically elevated in the basal ganglia and thalamus on SUVR maps (**f**, **g**). On the Z-score map, the ^18^F-THK-5351 signals on the bilateral basal ganglia and thalamus were markedly suppressed and the stroke-induced ^18^F-THK-5351 uptake was better visualized (**h**)

**Fig. 2:**
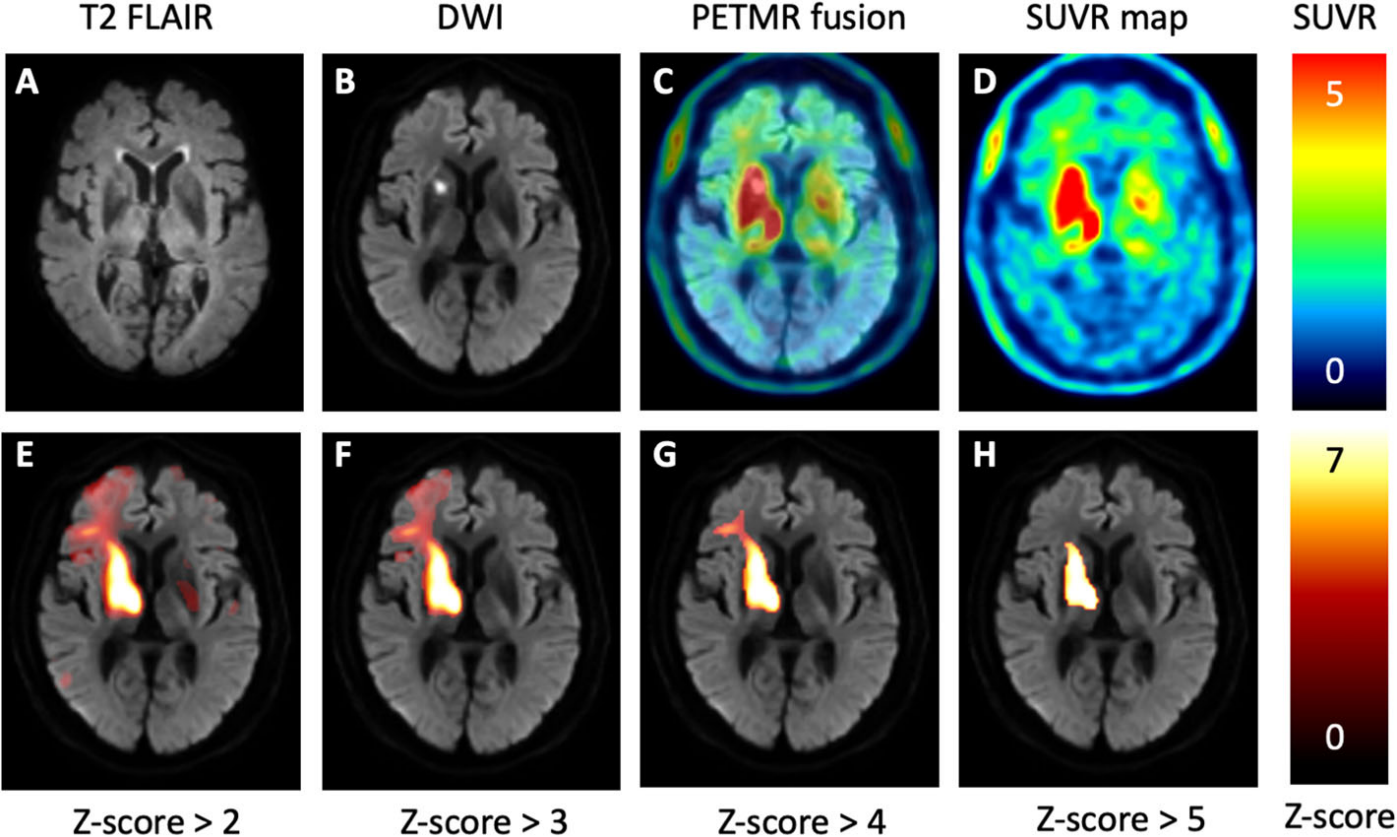
Comparisons of ^18^F-THK-5351 uptake patterns among the SUVR map and Z-maps at multiple Z-score thresholds. A patient with a lacunar infarct at the right internal capsule (**a**, **b**) shows increased ^18^F-THK-5351 uptake around the stroke lesion on the SUVR maps (**c**, **d**). On the Z-maps with sequentially increasing thresholds, the ^18^F-THK-5351 uptake regions gradually shrink (**e**–**h**). Furthermore, the increased ^18^F-THK-5351 uptake might extend to the cerebral cortex without corresponding changes observed on the conventional FLAIR and DWI images

To reduce the spurious uptake and registration error, each spatially normalized ^18^F-THK-5351 normal image was smoothed using a 3D Gaussian kernel of 8 mm full width at half maximum (FWHM). Then two normal ^18^F-THK-5351 SUVR images of mean (μ^TP^) and standard deviation (σ^TP^) were calculated from the smoothed and spatially normalized SUVR images of the healthy subjects. With voxelwise comparison to the mean and standard deviation values obtained above, the Z-score image for each subject with SUVR image *I* was finally computed as $Z=\frac{I-{\upmu}^{TP}}{\upsigma^{TP}}$. Through the transformation, we prominently diminished the background signals in the striatum and thalamus of patients with stroke (Fig. 1). Additionally, we built the ^18^F-THK-5351 Z-maps for patients with stroke at multiple Z-score thresholds; namely, *Z* > 2, *Z* > 3, *Z* > 4, and *Z* > 5 (Fig. 2). To investigate the correlation of stroke-induced ^18^F-THK-5351 uptake with PSCI, we calculated the Z-SUM scores at each Z-map threshold by summing the Z-scores within the whole brain (total Z-SUM scores), ipsilateral cerebral hemisphere (ipsilateral Z-SUM scores), and contralateral cerebral hemisphere (contralateral Z-SUM scores). In addition, we also calculated the Z-SUM scores in the stroke core region and the perilesional region.

### Statistical analyses

For descriptive statistics, we performed the two-sample *t* test, Chi-Square test, and Fisher’s exact test for group comparisons. Further, we performed Pearson’s correlation analysis to investigate the correlations of the Z-SUM scores of ^18^F-THK-5351 uptake intensity with demographic, vascular, and neurodegenerative factors. Moreover, we analyzed the partial correlations of cognitive performance with Z-SUM scores and other relevant factors after adjusting for age and education co-variables.

Since PSCI is associated with multiple factors, we employed multiple regression procedures using the forward selection method to determine the cognitive influence of total Z-SUM scores in three models. In model I, we adopted age and education as confounding factors. In model II, we added vascular factors, including NIHSS, stroke volume, and leukoaraiosis, as explanatory variables. In model III, neurodegenerative factors were further included. We applied 4 thresholds of ^18^F-THK-5351 Z-score (*Z* > 2, *Z* > 3, *Z* > 4, and *Z* > 5) in all the above correlation and regression analyses to determine the most sensitive cutoff level. Subsequently, we applied path analyses to evaluate whether Z-SUM scores of ^18^F-THK-5351 uptake intensity mediated the associations between stroke volume and PSCI severity after adjusting for age and education and mood conditions from NPI items (Sup. Fig. 1). Statistical analyses were performed with SAS version 9.0 (SAS Institute Inc., New York, USA), and *P* value < 0.05 was considered significant.

## Results

We enrolled 63 amyloid-negative, right-handed patients around 3 months after the first-ever index stroke (median 86 days with interquartile range from 73 to 98 days), and 25 of them had PSCI. Patients with PSCI had older age, lower education, and more severe cortical atrophy (Table 1), and also had moderate higher stroke volume, NIHSS, DWML, and MTA scores. Further, there was a moderate to significant difference in the whole brain ^18^F-THK-5351 uptake intensity between patients with and without PSCI at multiple threshold levels, including total Z-SUM-2 (*Z* > 2), Z-SUM-3 (*Z* > 3), and Z-SUM-4 (*Z* > 4) scores, but not total Z-SUM-5 (*Z* > 5) score. Besides, there was no difference in the presence of enlarged perivascular space, cerebral microbleeds, and lacunes between patients with and without PSCI. In addition, there was no difference in Z-SUM scores in terms of hypertension, diabetes mellitus, dyslipidemia, and current stroke habit for stroke patients and healthy subjects (Sup. Table 2).

**Table 1:** Clinical characteristics of patients with and without post-stroke cognitive impairment

Characteristics	Mean (SD)		
	Without PSCI (*n* = 38)	With PSCI (*n* = 25)	*P* value
Age, year	61.1 (7.1)	68.4 (9.4)	< 0.01
Education, year	10.4 (3.1)	7.5 (4.6)	< 0.01
Male, No. (%)	31 (82)	16 (64)	0.12
APOE ε4 carrier, No. (%)	4 (11)	3 (12)	1.00^a^
Days between stroke onset and cognition evaluation	102.4 (29.7)	101.6 (21.0)	0.91
Days between stroke onset and ^18^F-THK-5351 scanning	92.8 (14.8)	104.4 (24.4)	0.04
Days between stroke onset and ^18^F-florbetapir scanning	100.4 (31.6)	101.2 (28.4)	0.92
Common vascular risk factors			
Hypertension, No. (%)	31 (82)	24 (96)	0.13^a^
Diabetes mellitus, No. (%)	14 (37)	6 (24)	0.28
Dyslipidemia, No. (%)	32 (84)	17 (68)	0.13
Gout, No. (%)	7 (18)	3 (12)	0.72^a^
PVL score	0.4 (0.8)	0.8 (1.1)	0.13
DWML score	2.6 (1.3)	3.2 (1.2)	0.08
Enlarged perivascular space, No. (%)	11 (29)	8 (32)	0.80
Lobar microbleed, No. (%)	6 (16)	6 (24)	0.52^a^
Deep microbleed, No. (%)	6 (16)	2 (8)	0.46^a^
Lacune, No. (%)	10 (26)	4 (16)	0.34
Stroke features			
NIHSS	1.5 (1.3)	2.4 (2.3)	0.06
Ischemic stroke, No. (%)	35 (92)	23 (92)	1.00^a^
Left hemisphere stroke, No. (%)	17 (45)	15 (60)	0.24
Supratentorial stroke lesion, No. (%)	35 (92)	23 (92)	1.00^a^
Stroke volume, %	3.39E-6 (4.45E-6)	6.18E-6 (6.57E-6)	0.07
MTA score	0.8 (1.0)	1.3 (1.3)	0.09
Cortical thickness, mm	2.44 (0.08)	2.37 (0.10)	0.01
Total Z-SUM score at different Z levels			
Total Z-SUM-2, *Z* > 2	86763 (96363)	136773 (126302)	0.09
Total Z-SUM-3, *Z* > 3	43536 (58763)	82344 (82885)	0.03
Total Z-SUM-4, *Z* > 4	25822 (40406)	50558 (53591)	0.04
Total Z-SUM-5, *Z* > 5	17404 (31432)	31059 (34631)	0.11

*APOE ε4* apolipoprotein E ε4, *DWML* deep white matter leukoaraiosis, *MTA* medial temporal atrophy, *NIHSS* National Institutes of Health Stroke Scale, *PSCI* post-stroke cognitive impairment, *PVL* periventricular leukoaraiosis, *Z-SUM* sum of ^18^F-THK-5351 uptake intensity Z scores

Unless otherwise indicated, data are expressed as mean (SD)

^a^Analyzed by Fisher’s exact test

### Associations of ^18^F-THK-5351 uptake intensity with stroke features and cognition

While the total Z-SUM scores of ^18^F-THK-5351 uptake intensity significantly increased with stroke volume at all thresholds, there was no correlation of total Z-SUM scores with age, education, NIHSS, leukoaraiosis, enlarged perivascular space, cerebral microbleeds, lacunes, brain atrophy factors, and days from stroke onset at most of the Z-score thresholds (Sup. Table 3). The total Z-SUM scores were also significantly correlated with the Montreal Cognitive Assessment (MoCA), Instrumental Activities of Daily Living (IADL), Informant Questionnaire on Cognitive Decline in the Elderly (IQCODE), sum of boxes of Clinical Dementia Rating (CDR-SOB), as well as general cognitive, visuospatial, executive, and language functions, at multiple threshold levels (Table 2). Moreover, there were significant correlations of most cognitive results with other demographic, vascular, and neurodegenerative factors.

**Table 2:** Factors associated with cognitive performance

	^18^F-THK-5351 uptake intensity				Demographic factors		Vascular factors				Neurodegenerative factors	
	Total Z-SUM-2	Total Z-SUM-3	Total Z-SUM-4	Total Z-SUM-5	Age, year	Education, year	NIHSS	Stroke volume, %	PVL score	DWML score	MTA score	Cortical thickness, mm
MoCA	− 0.26*	− 0.29*	− 0.28*	− 0.22	− 0.42***	0.59***	− 0.55***†	− 0.23†	− 0.23	− 0.32*†	− 0.11	0.55***†
NPI	0.21	0.21	0.21	0.17	0.08	− 0.16	0.58***†	0.28*†	0.06	0.19	− 0.10	− 0.32†
Depressive symptoms^a^	0.07	0.05	0.04	0.01	0.06	− 0.11	0.62***†	0.19	0.25	0.22	− 0.06	− 0.24
Anxiety^b^	− 0.10	− 0.13	− 0.13	− 0.13	0.12	− 0.31*	0.28*	− 0.10	0.13	− 0.03	0.13	− 0.20
IADL	0.37**†	0.41***†	0.39**†	0.31*†	0.31*	− 0.47***	0.58***†	0.29*†	0.12	0.19	0.03	− 0.44***
IQCODE^c^	0.42***†	0.42***†	0.37**†	0.29*†	0.34**	− 0.25	0.42***†	0.4**†	0.24	0.43***†	0.19	− 0.35**
CDR-SOB	0.31*	0.36**†	0.35**†	0.27*†	0.35**	− 0.32**	0.57***†	0.29*†	0.18	0.23	0.03	− 0.45***†
Composite cognitive *z* score												
General cognitive function	− 0.28*	− 0.36**†	− 0.38**†	− 0.34**†	− 0.26*	0.51***	− 0.43***†	− 0.31*†	− 0.24	− 0.29*†	− 0.15	0.39**
Memory function	− 0.11	− 0.16	− 0.18	− 0.15	− 0.35**	0.36**	− 0.37**†	− 0.14	− 0.38**†	− 0.27*	− 0.18	0.36**
Visuospatial function	− 0.28*	− 0.28*	− 0.27*	− 0.23	− 0.30*	0.21	− 0.25	− 0.24†	− 0.09	− 0.26*	0.12†	0.26*
Executive function	− 0.34**†	− 0.44***†	− 0.45***†	− 0.43***†	− 0.09	0.39**	− 0.41***†	− 0.36**†	− 0.06	− 0.21	− 0.10	0.32**
Language function	− 0.23	− 0.29*†	− 0.32*†	− 0.31*†	− 0.12	0.66***	− 0.25	− 0.28*†	− 0.09	− 0.22†	− 0.14	0.24

*CDR* clinical dementia rating, *DWML* deep white matter leukoaraiosis, *IADL* instrumental activities of daily living, *IQCODE* informant questionnaire on cognitive decline in the elderly, *MoCA* Montreal cognitive assessment, *MTA* medial temporal atrophy, *NIHSS* National Institutes of Health Stroke Scale, *NPI* neuropsychiatric inventory, *PVL* periventricular leukoaraiosis, *SOB* sum of boxes, *Z-SUM* sum of ^18^F-THK-5351 uptake intensity Z scores

*P < 0.05; **P < 0.01; ***P < 0.001; †*P* < 0.05 after adjustment for age and education

^a^Evaluated by the NPI depression item 4

^b^Evaluated by the NPI anxiety item 5

^C^Performed around 3 months after stroke

^18^F-THK-5351 Z-SUM scores were further calculated in the stroke core and perilesional regions (Table 3). The Z-SUM scores of stroke core and perilesional regions were both correlated with stroke volume, but not with NIHSS. Furthermore, the cognitive performance was associated with the perilesional Z-SUM scores rather than the stroke core Z-SUM scores after age and education adjustment.

**Table 3:** Correlations of Z-SUM scores of the stroke core and perilesional regions with stroke severity, stroke volume, and cognitive performance

	Stroke core regions					Perilesional regions		
	Z-SUM-2	Z-SUM-3	Z-SUM-4	Z-SUM-5	Z-SUM-2	Z-SUM-3	Z-SUM-4	Z-SUM-5
NIHSS	0.18	0.17	0.14	0.10	0.22	0.24	0.19	0.14
Stroke volume, %	0.73***†	0.69***†	0.63***†	0.56***†	0.41***†	0.51***†	0.55***†	0.54***†
MoCA	− 0.13	− 0.12	− 0.09	− 0.05	− 0.26*	− 0.29*	− 0.29*	− 0.24
NPI	0.14	0.12	0.09	0.05	0.20	0.21	0.22	0.19
Depressive symptoms^a^	0.05	0.04	0.00	− 0.04	0.07	0.05	0.04	0.02
Anxiety^b^	− 0.10	− 0.10	− 0.10	− 0.10	− 0.09	− 0.13	− 0.13	− 0.13
IADL	0.19	0.18	0.15	0.12	0.37**†	0.41***†	0.41**†	0.33**†
IQCODE^c^	0.14	0.11	0.07	0.02	0.42***†	0.44***†	0.40**†	0.33**†
CDR-SOB	0.15	0.13	0.11	0.08	0.31*	0.37**†	0.37**†	0.29*†
Composite cognitive *z* score								
General cognitive function	− 0.26*	− 0.25*	− 0.23	− 0.21	− 0.27*	− 0.35**†	− 0.38**†	− 0.35**†
Memory function	− 0.12	− 0.11	− 0.10	− 0.09	− 0.11	− 0.16	− 0.18	− 0.15
Visuospatial function	− 0.18	− 0.18	− 0.17	− 0.16	− 0.28*	− 0.28*	− 0.27*	− 0.23
Executive function	− 0.28*	− 0.27*	− 0.25	− 0.21	− 0.33**†	− 0.43***†	− 0.46***†	− 0.44***†
Language function	− 0.31*	− 0.31*	− 0.30*	− 0.27*	− 0.22	− 0.28*†	− 0.31*†	− 0.30*†

*CDR* clinical dementia rating, *IADL* instrumental activities of daily living, *IQCODE* informant questionnaire on cognitive decline in the elderly, *MoCA* Montreal cognitive assessment, *NIHSS* National Institutes of Health Stroke Scale, *NPI* neuropsychiatric inventory, *SOB* sum of boxes, *Z*-*SUM* sum of ^18^F-THK-5351 uptake intensity Z scores

*P < 0.05; **P < 0.01; ***P < 0.001; †*P* < 0.05 after adjustment for age and education

^a^Evaluated by the NPI depression item 4

^b^Evaluated by the NPI anxiety item 5

^c^Performed around 3 months after stroke

**Fig. 3:**
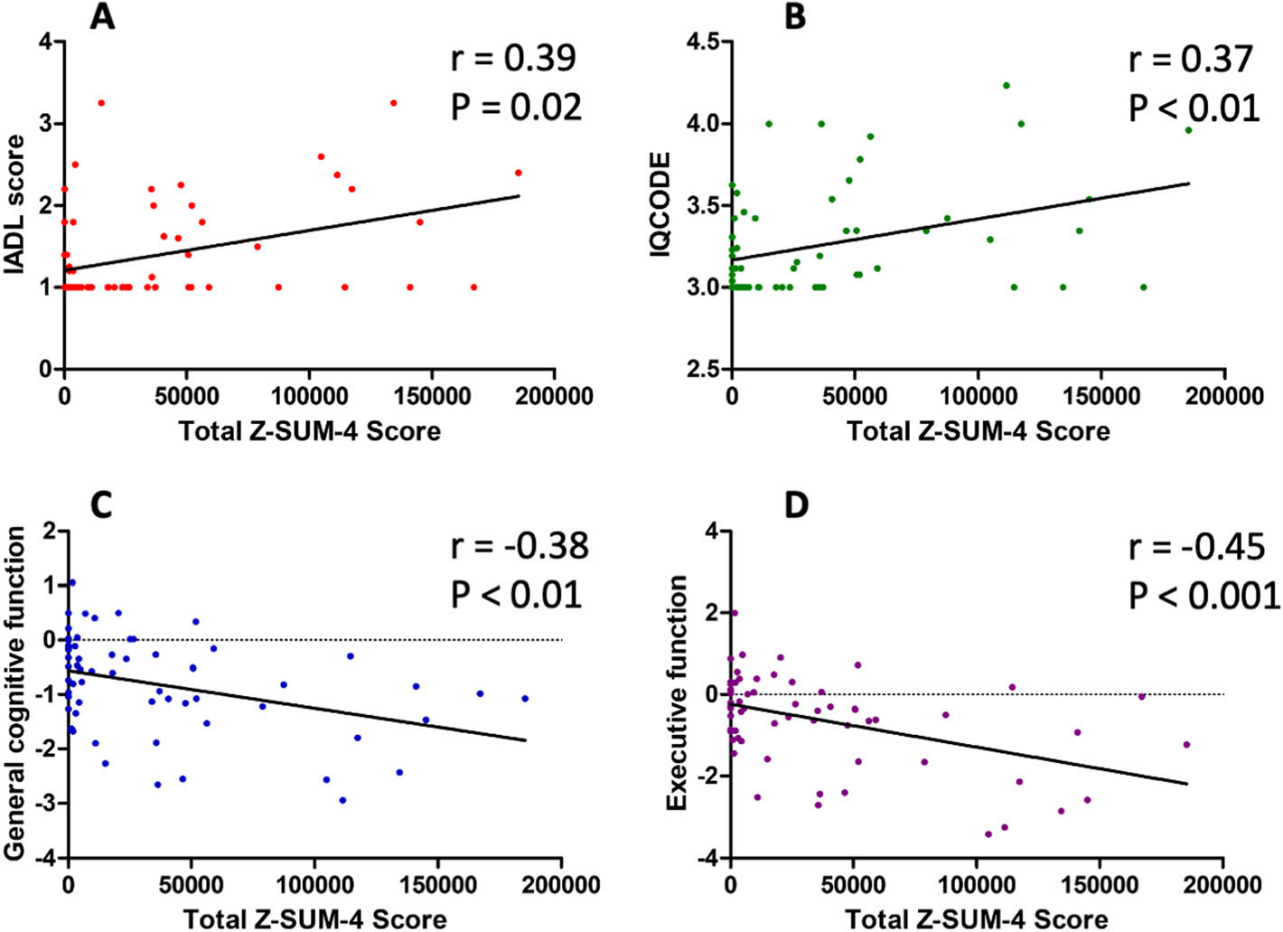
The correlations of cognitive results with total Z-SUM-4 scores, the normalized ^18^F-THK-5351 uptake intensity. The total Z-SUM-4 score was significantly correlated with Instrumental Activities of Daily Living (IADL) score (**a**), Informant Questionnaire on Cognitive Decline in the Elderly (IQCODE) score (**b**), general cognitive function (**c**), and executive function performance (**d**)

Given the complex associations among demographic data, vascular imaging findings, brain atrophy, and cognition, we built three multiple linear regression models to decipher the influence of ^18^F-THK-5351 uptake intensity on post-stroke cognitive performance. In model I, age and education were taken as the co-variables (Sup. Table 4). Vascular factors, including NIHSS, stroke volume, and leukoaraiosis, were added as explanatory variables in model II (Sup. Table 5); neurodegenerative factors were further included in model III (Sup. Table 6). Among the three models, there was a similar trend of associations between total Z-SUM scores of ^18^F-THK-5351 and cognitive performance, especially for IADL, IQCODE, general cognitive and executive functions, and the total Z-SUM-4 score was most sensitive to cognitive changes (Fig. 3).

### Stroke lesion side stratification

To investigate the influence of stroke lesion side on PSCI, we excluded 5 patients with infratentorial stroke lesions from the analyses. The correlation coefficients of stroke volume with ipsilateral Z-SUM-2 to Z-SUM-5 scores were 0.48, 0.54, 0.56, and 0.54 (*P*s < 0.01), while the correlation coefficients of stroke volume with contralateral Z-SUM-2 to Z-SUM-5 scores were 0.23 (*P* = 0.08), 0.26 (*P* = 0.04), 0.23 (*P* = 0.08), and 0.19 (*P* = 0.16). Moreover, the correlation coefficients between ipsilateral and contralateral hemisphere Z-SUM scores were 0.64 (*P* < 0.01), 0.39 (*P* < 0.01), 0.13 (*P* = 0.35), and 0.02 (*P* = 0.90) for Z thresholds from 2 to 5.

We further stratified patients into left- and right-hemispheric stroke. There was no significant difference in the demographic data, imaging findings, and cognitive results between patients with right- and left-hemispheric stroke (Sup. Table 7). The total Z-SUM scores were significantly correlated with CDR-SOB, general cognitive function, and each cognitive domain function in patients with left-hemispheric stroke and with executive function in patients with right-hemispheric stroke after adjustment for age and education. Furthermore, cognitive performance was more prominently correlated with the ipsilateral Z-SUM scores (Table 4), but not with the contralateral Z-SUM scores (Sup. Table 8). With regard to stroke volume, it was correlated with general cognitive and language functions in patients with left-hemispheric stroke, and with IQCODE, CDR-SOB, and general cognitive and executive functions in patients with right-hemispheric stroke.

**Table 4:** Correlations of Z-SUM scores with cognitive performance in patients with left and right hemisphere stroke

	MoCA	IADL	IQCODE^a^	CDR-SOB	General cognitive function	Memory	Visuospatial function	Executive function	Language
Patients with left hemisphere stroke (*n* = 30)									
Stroke volume,%	− 0.24	0.25	0.33	0.13	− 0.36†	− 0.15	− 0.19	− 0.34	− 0.56*
Total Z-SUM-2	− 0.41*	0.46*	0.40*	0.45*†	− 0.48*†	− 0.38*	− 0.41*†	− 0.40*	− 0.42*
Total Z-SUM-3	− 0.44*†	0.48*†	0.34	0.49*†	− 0.54*†	− 0.39*†	− 0.36†	− 0.49*†	− 0.51*†
Total Z-SUM-4	− 0.41*†	0.41*	0.24	0.46*†	− 0.52*†	− 0.36†	− 0.30†	− 0.48*†	− 0.52*†
Total Z-SUM-5	− 0.32†	0.32	0.16	0.36*†	− 0.44*†	− 0.29†	− 0.20	− 0.43*†	− 0.48*†
Ipsilateral Z-SUM-2	− 0.57*†	0.55*†	0.41*†	0.55*†	− 0.57*†	− 0.42*†	− 0.46*†	− 0.50†	− 0.52†
Ipsilateral Z-SUM-3	− 0.56*†	0.54*†	0.34	0.55*†	− 0.59*†	− 0.40*†	− 0.40*†	− 0.55*†	− 0.56*†
Ipsilateral Z-SUM-4	− 0.51*†	0.47*†	0.25	0.50*†	− 0.55*†	− 0.36†	− 0.32†	− 0.53*†	− 0.56*†
Ipsilateral Z-SUM-5	− 0.40*†	0.37*†	0.15	0.39*†	− 0.47*†	− 0.28†	− 0.22†	− 0.47*†	− 0.52*†
Patients with Right Hemisphere Stroke (*n*= 28)									
Stroke volume, %	− 0.27	0.30	0.43*†	0.41*†	− 0.33†	− 0.20	− 0.37	− 0.44*†	0.01
Total Z-SUM-2	− 0.14	0.23	0.39*	0.20	− 0.09	0.08	− 0.17	− 0.27	− 0.05
Total Z-SUM-3	− 0.15	0.29	0.45*	0.25	− 0.19	0.01	− 0.20	− 0.38*†	− 0.07
Total Z-SUM-4	− 0.13	0.32	0.46*	0.26	− 0.22	− 0.01	− 0.24	− 0.42*†	− 0.08
Total Z-SUM-5	− 0.07	0.26	0.38*	0.18	− 0.21	0.00	− 0.28	− 0.41*†	− 0.07
Ipsilateral Z-SUM-2	− 0.16	0.28	0.43*	0.19	− 0.16	0.06	− 0.25	− 0.37†	− 0.08
Ipsilateral Z-SUM-3	− 0.16	0.32	0.47*	0.24	− 0.22	0.00	− 0.25	− 0.44*†	− 0.09
Ipsilateral Z-SUM-4	− 0.13	0.33	0.46*	0.26	− 0.23	− 0.02	− 0.25	− 0.44*†	− 0.08
Ipsilateral Z-SUM-5	− 0.07	0.26	0.39*	0.18	− 0.21	0.00	− 0.28	− 0.41*†	− 0.07

*CDR* clinical dementia rating, *IADL* instrumental activities of daily living, *IQCODE* informant questionnaire on cognitive decline in the elderly, *MoCA* Montreal cognitive assessment, *NIHSS* National Institutes of Health Stroke Scale, *NPI* neuropsychiatric inventory, *SOB* sum of boxes, *Z*-*SUM* sum of ^18^F-THK-5351 uptake intensity Z scores

*P < 0.05; † *P* < 0.05 after adjustment for age and education

^a^Performed around 3 months after stroke

**Fig. 4:**
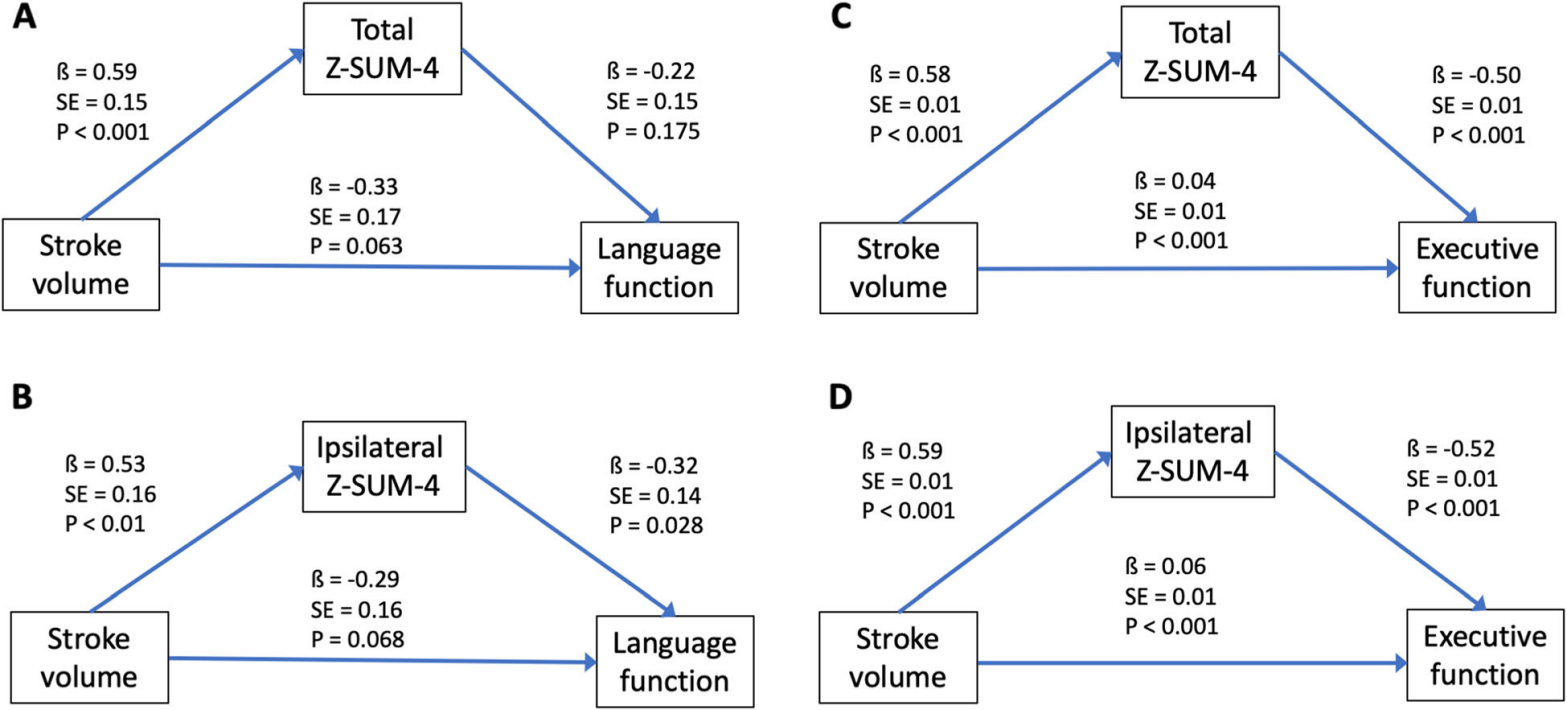
Path analyses of stroke effects on cognition via total or ipsilateral Z-SUM-4 scores after age, education and depressive symptoms adjustment. Stroke volume contributes significantly to cognitive function as well as total and ipsilateral Z-SUM-4 scores in patients with either left-hemispheric (**a**, **b**) or right-hemispheric (**c**, **d**) stroke. Further, ipsilateral Z-SUM-4 scores can partly mediate language influence from stroke volume in patients with left-hemispheric stroke (**b**). Likewise, total and ipsilateral Z-SUM-4 scores can partly mediate executive influence from stroke volume in patients with right-hemispheric stroke (**c**, **d**)

We conducted path analyses to explore the mediation effects of Z-SUM scores on the associations between stroke volume and PSCI severity after adjusting for age, education, and depressive symptoms (Fig. 4) and anxiety (Sup. Fig. 2) covariables (Sup. Table 9). We adopted the Z-SUM-4 score as the mediator because it was most sensitive to cognitive changes based on the above regression model results. We selected cognitive tests both correlated with stroke volume and Z-SUM-4 scores as the endogenous variables. Path analyses showed that stroke volume had significant direct effects on the total and ipsilateral Z-SUM-4 scores in patients with either left or right hemisphere stroke. In addition to the direct cognitive influence from stroke volume, ipsilateral Z-SUM-4 scores also significantly mediated the associations between stroke volume and language function in patients with left-hemispheric stroke and between stroke volume and executive function in patients with right-hemispheric stroke, respectively.

## Discussion

PSCI is usually presented in patients 3 to 6 months after stroke onset. A previous study suggested that neuroinflammation plays a role in the modulation of post-stroke cognitive performance [35]. In this study, we built an ^18^F-THK-5351 PET imaging template using data obtained from amyloid-negative healthy subjects to diminish background signals in the basal ganglia, thalamus, and brainstem. We enrolled amyloid-negative patients with first-ever stroke to investigate the RA extent, which was defined as the Z-SUM scores on ^18^F-THK-5351 PET imaging. Patients with PSCI had higher total Z-SUM scores of ^18^F-THK-5351 uptake intensity than those without PSCI. The total and ipsilateral Z-SUM scores were also associated with the patterns of post-stroke cognitive performance. Furthermore, path analyses indicated that the influence of stroke events on PSCI resulted from stroke volume itself as well as the RA extent indicated as the Z-SUM scores. This is the first study to demonstrate the associations between PSCI and RA extent on ^18^F-THK-5351 PET imaging.

We observed increased ^18^F-THK-5351 uptake intensity on Z-maps around the stroke lesion, which faded with distance away from the lesion core. This uptake distribution pattern could mimic the scar-like structures resulting from the proliferation and migration of reactive astrocytes in the ischemic penumbra [36, 37]. These scar-forming astrocytes have been reported to separate nonfunctional, nonneural lesion core tissue from immediately surrounding and potentially functional neural tissue [13]. Although astrocytes may produce scaffolding proteins to guide neuron growth [38], astrocyte scars have been widely regarded as a major impediment to axon regeneration after CNS injury [39]. In addition, disrupted glioneuronal interaction can cause synaptic dysfunction and cognitive impairment [40].

The cognitive influence from stroke lesions not only results from the focal factors, such as stroke volume and location, but also can be attributed to the perilesional and remote effects of these lesions [41]. Previous diffusion tensor imaging studies have reported that changes in perilesional and remote areas had a greater influence on cognitive manifestations than the stroke lesion core itself [42, 43]. Similarly in our study, the perilesional Z-SUM scores rather than the stroke core Z-SUM scores were associated with cognitive performance. In addition, we also stratified the Z-SUM scores into total, ipsilateral, and contralateral scores, and we found ipsilateral Z-SUM scores tended to have more correlation with cognitive performance than total Z-SUM scores. Future multi-modality imaging studies could employ in vivo RA visualization on PET images to investigate the neuroinflammation influence on perilesional and remote areas via structural and functional connectivity analyses, which might help understand the pathophysiological bases of PSCI [41].

In our study, we applied the Z-map method to contrast the stroke-induced RA extent on ^18^F-THK-5351 PET imaging, which may mimic the inflammatory penumbra [44, 45]. As inflammation is a common reaction to miscellaneous physical conditions, applying a threshold to acquire the Z-SUM scores would help to filter out signals not induced by stroke lesion. We found the Z-SUM-4 scores (with values at least 4 standard deviations higher than the mean at each voxel) were more sensitive to PSCI severity.

Post-stroke neuroinflammation is a diffuse process. In our study, we explored the associations of bilateral hemisphere RA with stroke volume and cognition. We found that stroke volume was significantly correlated with ipsilateral Z-SUM scores, but its correlations with contralateral Z-SUM scores were relatively minor at multiple *Z* thresholds (*Z* > 2, *Z* > 3, and *Z* > 4). Further, the correlations between bilateral cerebral hemispheric Z-SUM-2 and Z-SUM-3 scores were significant. Besides, we also found the contralateral Z-SUM scores of stroke patients were higher than the average Z-SUM scores of bilateral cerebral hemispheres of the 22 healthy controls (at *Z* > 2, *Z* > 3, and *Z* > 4 thresholds; Sup. Table 10). However, the Z-SUM scores in the contralateral hemisphere were not associated with cognitive performance. These findings suggest that post-stroke neuroinflammation is a diffuse process, which is more prominent in the ipsilateral hemisphere and minor in the contralateral hemisphere.

^18^F-THK-5351 has the alternative affinity for monoamine oxidase-B (MAO-B), and MAO-B is largely expressed in astrocytes in response to CNS damage [12, 13]. We took the advantage of such characteristics to explore RA presentations in first-ever stroke patients. In our study, we adopted several major steps to decrease the possibility of cross-binding of ^18^F-THK-5351 to tau protein. Firstly, we included amyloid-negative first-ever stroke patients to decrease the possibility of co-existing AD. Secondly, we recruited stroke patients without medical history and imaging results suggestive of tauopathy, including AD, progressive supranuclear palsy, corticobasal degeneration, and fronto-temporal dementia, and the patient recruitment was evaluated after consensus by a multidiscipline team. Furthermore, as tau protein has been reported as a marker of axonal injury, ischemic stroke may induce a transient tau protein increase in human CSF, with a peak 1 week after stroke onset and a normalization after 3 months [46, 47]. In our study, we recruited patients around 3 months after stroke to decrease the acute stroke-related effect on tau protein formation. Although the transformed ^18^F-THK-5351 uptake values may be more relevant to stroke-induced RA and appear correlated with PSCI after these procedures, the actual accuracy and sensitivity of ^18^F-THK-5351 to RA in stroke patients need to be clarified in future studies. Further pathological and neuroimaging investigations could provide more direct evidence on the interaction between stroke-induced RA and PSCI.

Both acute ischemic stroke and hemorrhagic stroke are associated with glial toxicity and cell injury, but with different spatio-temporal neuroinflammatory processes and mechanisms [48, 49]. Although astrocytes have differential roles in the recovery patterns of ischemic and hemorrhagic stroke, the long-term GFAP-positive astrocytic plasticity could be similar after both ischemic and hemorrhagic stroke [50, 51]. In our sub-analyses, the associations between Z-SUM scores and PSCI presentations were significant in ischemic patients, and the trends were similar to the results of the pooled population (Sup. Table 11). The sample size of patients with hemorrhagic stroke (*n* = 5) was insufficient to investigate such associations. As there was no difference in clinical characteristics, stroke volume, and Z-SUM scores between patients with ischemic and hemorrhagic stroke, we pooled these two types of patients together to explore the influence of RA on PSCI (Sup. Table 12). However, RA response to ischemic and hemorrhagic stroke may be dynamic and versatile in the recovery process, and further longitudinal studies are warranted to investigate the RA influence on PSCI presentations in different stroke subtypes.

Patients with stroke are more susceptible to attention, spatial ability, language, and executive function impairments rather than memory problem [21]. These findings were in line with our study result that stroke volume and total Z-SUM scores of ^18^F-THK-5351 were correlated with most of cognitive results, but not with memory performance (Table 2). Education attainment is associated with PSCI severity, and we have taken education into account when investigating the associations between RA extent and PSCI performance in the correlation, regression, and path analyses. Other risk factors, such as age, stroke volume, as well as hippocampal and cortical atrophy, are associated with the incidence of PSCI [1–4, 52]. Similar findings were also noted in our study. After adjusting for confounding factors in the multiple regression analyses, the total Z-SUM scores were still correlated with IADL, IQCODE, and the general cognitive and executive functions in most models.

Both PSCI and post-stroke depression are associated with late worsening of disability, and an immunological hypothesis is one of the mechanisms of these two stroke sequelae [51]. Besides, post-stroke depression could also be a potential source of PSCI. In our study, there was no difference in depressive symptoms and anxiety between patients with and without PSCI, and the influence of RA extent on cognitive performance remained significant after adjusting for these mood conditions in path analyses. As the etiology of post-stroke depression remains controversial, further studies are necessary to investigate the associations between neuroimaging and fluid inflammatory biomarkers associated with post-stroke depression [53].

Left-hemispheric stroke is reported as an important risk factor for PSCI [54]. Similar findings were noted in our study that patients with left hemisphere stroke tended to score lower in each cognitive domain battery than patients with right hemisphere stroke (Sup. Table 7). When focusing on the cognitive influence of stroke-induced RA on specific cerebral hemisphere, ipsilateral Z-SUM scores were correlated with most cognitive results in patients with left hemisphere stroke, while ipsilateral Z-SUM scores were correlated with IQCODE and executive function in patients with right hemisphere stroke (Table 4). Furthermore, under path analyses, PSCI severity could be attributed to stroke lesion volume directly as well as stroke-induced inflammation indirectly in patients with right or left hemisphere stroke, suggesting that stroke-induced RA may have a modulating effect on PSCI occurrence (Sup. Table 9).

Currently, other radioligands targeting the neuroinflammatory process are under development. Microglia can be imaged using the ^11^C-PK11195 and ^11^C-PBR28 radiotracers, which have a high affinity for the 18-kDa translocator protein found in the mitochondria of microglia. Post-stroke microglia activation has been investigated using ^11^C-PK11195 PET imaging, but there is limited literature on the relationship between ^11^C-PK11195 findings and PSCI [9, 55]. In contrast, the ^11^C-deuterium-l-deprenyl (^11^C-DED) and ^18^F-SMBT-1 radiotracers are designed to bind to MAO-B in astrocytes [5]. The utility of ^11^C-DED radiotracer is limited by the short half-life of carbon-11 and their suboptimal binding specificity [56]. The ^18^F-SMBT-1 radiotracer is a derivative of the ^18^F-THK-5351 compound, and has shown high specificity to astrogliosis in an animal study recently [57]. The application of ^18^F-SMBT-1 PET in stroke patients may further substantiate the role of RA in PSCI occurrence.

This study had several limitations. First, although ^18^F-THK-5351 can be used to quantify the RA extent in patients with stroke through the Z-map transformation method, there is still a need to validate the optimal Z-score threshold for demonstrating the RA extent. Our preliminary findings indicated that the Z-score > 4 threshold was most sensitive to PSCI manifestations. Second, ^18^F-THK-5351 has a dual binding affinity to both tau protein and MAO-B, and such characteristic could limit the general applicability of ^18^F-THK-5351 in patients with stroke for neuroinflammation evaluation. Since our main interest was to explore the relationships between PSCI and neuroinflammation on ^18^F-THK-5351 PET imaging, we only enrolled amyloid-negative stroke patients without tauopathy presentations to reduce the possibility of ^18^F-THK-5351 binding to tau protein. The development of novel radiotracers specific to RA would be helpful to determine the cognitive impacts of stroke-related RA. Third, ^18^F-THK-5351 PET was performed about 3 months after stroke in our study. As neuroinflammation effects on tissue remodeling are dynamic after neuronal injury, the RA presentations on ^18^F-THK-5351 PET imaging in acute stroke stage are yet to be determined [13]. Finally, this was a cross-sectional study with relatively small sample size. Future long-term follow-up studies should further investigate the dynamic relationships between RA and PSCI manifestations.

## Conclusion

Stroke-induced ^18^F-THK-5351 uptake signals could reflect the RA extent in amyloid-negative patients with first-ever stroke. Besides the cognitive effect from stroke lesion itself, stroke-induced neuroinflammation as measured by the total and ipsilateral Z-SUM scores of ^18^F-THK-5351 PET imaging could further contribute to PSCI presentations.

## Supplementary information

**Additional file 1: Supplementary Table 1.** Tests for neuropsychological function and stroke severity evaluation

**Additional file 2: Supplementary Figure 1.** The illustration of path analyses models. We used path analyses to evaluate whether Z-SUM scores of ^18^F-THK-5351 uptake intensity mediated the associations between stroke volume and cognitive performance. We selected cognitive functions showing significant correlations with both stroke volume and Z-sum scores as the endogenous variables. Further, we adjusted for age, education, and depressive symptoms (a) and anxiety (b) co-variables.

**Additional file 3: Supplementary Table 2.** The comparisons of total Z-SUM scores between the presence and absence of vascular risk factors in stroke patients and healthy subjects

**Additional file 4: Supplementary Table 3.** Correlations of total Z-SUM scores with demographic, vascular and neurodegenerative features

**Additional file 5: Supplementary Table 4.** Associations of total Z-SUM scores of ^18^F-THK-5351 uptake intensity with cognitive function in model I

**Additional file 6: Supplementary Table 5.** Associations of total Z-SUM scores of ^18^F-THK-5351 uptake intensity with cognitive function in model II

**Additional file 7: Supplementary Table 6.** Associations of total Z-SUM scores of ^18^F-THK-5351 uptake intensity with cognitive function in model III

**Additional file 8: Supplementary Table 7.** Demographic data, imaging findings and cognitive results between patients with left and right hemisphere stroke

**Additional file 9: Supplementary Table 8.** Correlations of contralateral Z-SUM scores with cognitive performance in patients with left and right hemisphere stroke

**Additional file 10: Supplementary Figure 2.** Path analyses of stroke effects on cognition via total or ipsilateral Z-SUM-4 scores after age, education and anxiety adjustment. Stroke volume contributes significantly to cognitive function as well as total and ipsilateral Z-SUM-4 scores in patients with either left-hemispheric (a, b) or right-hemispheric (c, d) stroke. Further, ipsilateral Z-SUM-4 scores can partly mediate language influence from stroke volume in patients with left-hemispheric stroke (b). Likewise, total and ipsilateral Z-SUM-4 scores can partly mediate executive influence from stroke volume in patients with right-hemispheric stroke (c, d)

**Additional file 11: Supplementary Table 9.** Effects of stroke volume and Z-SUM-4 score on cognitive function after adjustment for age, education and depressive symptoms (A~D) and anxiety (E~H)

**Additional file 12: Supplementary Table 10.** Comparisons of the contralateral Z-SUM scores of stroke patients with the average Z-SUM scores of bilateral hemispheres of healthy subjects

**Additional file 13: Supplementary Table 11.** The correlations between total Z-SUM scores and cognitive function in ischemic stroke patients

**Additional file 14: Supplementary Table 12.** Clinical characteristics of patients with hemorrhagic and ischemic stroke

## Data Availability

The datasets used and/or analyzed during the current study are available from the corresponding author on reasonable request.

## Abbreviations

AD: Alzheimer’s disease
APOE ε4: Apolipoprotein E ε4
^11^C-DED: ^11^C-deuterium-l-deprenyl
CDR-SOB: Sum of boxes of Clinical Dementia Rating
CT: Computed tomography
DWI: Diffusion-weighted imaging
DWML: Deep white matter leukoaraiosis
FLAIR: Fluid-attenuated inversion recovery
FWHM: Full width at half maximum
IADL: Instrumental Activities of Daily Living
IQCODE: Informant Questionnaire on Cognitive Decline in the Elderly
MAO-B: Monoamine oxidase-B
MMSE: Mini-Mental State Examination
MoCA: Montreal Cognitive Assessment
MPRAGE: Magnetization Prepared RApid Gradient Echo
MRI: Magnetic resonance imaging
MTA: Medial temporal atrophy
NIHSS: National Institutes of Health Stroke Scale
NPI: Neuropsychiatric Inventory
PET: Positron-emission tomography
PSCI: Post-stroke cognitive impairment
PSD: Post-stroke dementia
PVL: Periventricular leukoaraiosis
RA: Reactive astrogliosis
SUVR: Standardized uptake value ratio
T1W: T1-weighted
Z-SUM scores: Sum of ^18^F-THK-5351 uptake intensity Z scores
μ^TP^: Mean
σ^TP^: Standard deviation
