# Supplementary material for: Investigation of reactive astrogliosis effect on post-stroke cognitive impairment
Source: J Neuroinflammation. 2020 Oct 17;17:308. doi: 10.1186/s12974-020-01985-0 (PMC7568828; doi:10.1186/s12974-020-01985-0)
Supplement: Supplementary file 1 — Additional file 1: Supplementary Table 1. Tests for neuropsychological function and stroke severity evaluation [file 12974_2020_1985_MOESM1_ESM.docx]

| **Supplementary Table 1.** Tests for neuropsychological function and stroke severity evaluation |
| --- |
| Neuropsychiatric screening |
| Montreal Cognitive Assessment (MoCA) |
| Instrumental Activities of Daily Living (IADL)^a^ |
| Informant Questionnaire on Cognitive Decline in the Elderly (IQCODE)^b^ |
| Neuropsychiatric Inventory (NPI) |
|  |
| Memory domain evaluation |
| Immediate recall score of the California Verbal Learning Test (CVLT)-Chinese Version |
| Delayed recall score of the California Verbal Learning Test (CVLT)-Chinese Version |
| Delayed recognition score of the California Verbal Learning Test (CVLT)-Chinese Version |
| Immediate recall score of the Brief Visual Memory Test (BVMT) |
| Delayed recall score of the Brief Visual Memory Test (BVMT) |
| Delayed recognition score of the Brief Visual Memory Test (BVMT) |
|  |
| Visuospatial domain evaluation |
| Position Discrimination subtest of the Visual Object and Space Perception (VOSP) Battery |
| Number Location subtest of the Visual Object and Space Perception (VOSP) Battery |
|  |
| Executive domain evaluation |
| Design Fluency test of the Delis-Kaplan Executive System |
| Colored Trail Making Test-A and -B (TMT-A & TMT-B) |
|  |
| Language domain evaluation |
| Chinese Graded Word Reading Test (CGWRT) |
| Total score and switching score of the Category Verbal Fluency Test |
|  |
| Stroke severity evaluation |
| National Institutes of Health Stroke Scale (NIHSS) |
| ^a^ Since some items in the IADL were not scored among male patients, the average of all scored items was taken as the final IADL score. |
| ^b^ IQCODE was done twice; the first test was done within 1 week after stroke as one of the inclusion criteria for pre-stroke cognitive function evaluation, and the second test was done around 3 months after stroke for post-stroke cognitive impairment severity evaluation. |
