# Supplementary material for: Investigation of reactive astrogliosis effect on post-stroke cognitive impairment
Source: J Neuroinflammation. 2020 Oct 17;17:308. doi: 10.1186/s12974-020-01985-0 (PMC7568828; doi:10.1186/s12974-020-01985-0)

**Supplementary Figure 1.** The illustration of path analyses models. We used path analyses to evaluate whether Z-SUM scores of ^18^F-THK-5351 uptake intensity mediated the associations between stroke volume and cognitive performance. We selected cognitive functions showing significant correlations with both stroke volume and Z-sum scores as the endogenous variables. Further, we adjusted for age, education, and depressive symptoms (a) and anxiety (b) co-variables.


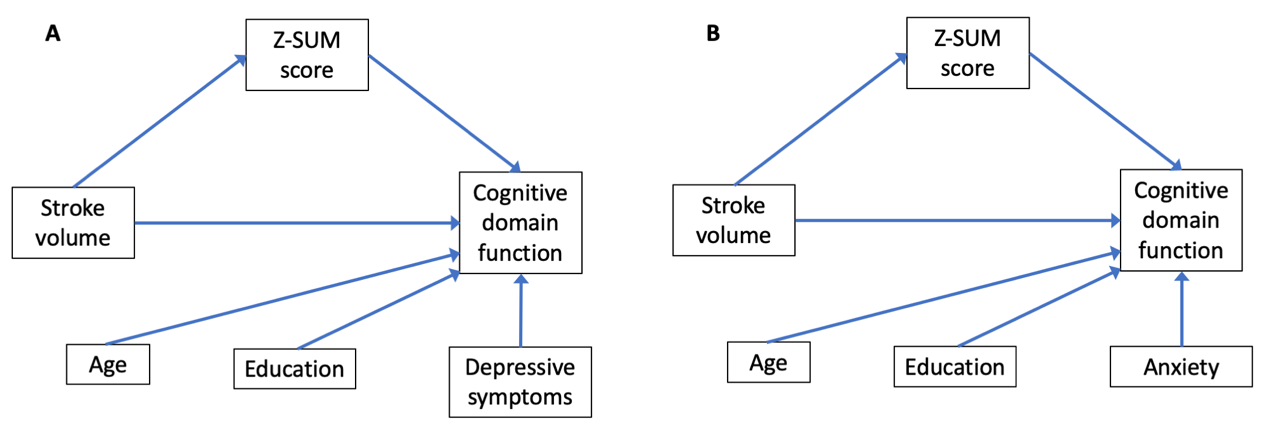

Supplement: Supplementary file 2 — Additional file 2: Supplementary Figure 1. The illustration of path analyses models. We used path analyses to evaluate whether Z-SUM scores of 18F-THK-5351 uptake intensity mediated the associations between stroke volume and cognitive performance. We selected cognitive functions showing significant correlations with both stroke volume and Z-sum scores as the endogenous variables. Further, we adjusted for age, education, and depressive symptoms (a) and anxiety (b) co-variables. [file 12974_2020_1985_MOESM2_ESM.docx]
