# Supplementary material for: Investigation of reactive astrogliosis effect on post-stroke cognitive impairment
Source: J Neuroinflammation. 2020 Oct 17;17:308. doi: 10.1186/s12974-020-01985-0 (PMC7568828; doi:10.1186/s12974-020-01985-0)
Supplement: Supplementary file 3 — Additional file 3: Supplementary Table 2. The comparisons of total Z-SUM scores between the presence and absence of vascular risk factors in stroke patients and healthy subjects [file 12974_2020_1985_MOESM3_ESM.docx]

| **Supplementary Table 2.** The comparisons of total Z-SUM scores between the presence and absence of vascular risk factors in stroke patients and healthy subjects | | | | | | | |
| --- | --- | --- | --- | --- | --- | --- | --- |
|  | Stroke patients | | |  | Healthy subjects | | |
|  | Hypertension | | |  | Hypertension | | |
|  | No (n = 8) | Yes (n = 55) | P value |  | No (n = 11) | Yes (n = 11) | P value |
| Total Z-SUM-2 score | 133978 (168831) | 103256 (101531) | 0.63 |  | 23430 (55670) | 6231 (7307) | 0.33 |
| Total Z-SUM-3 score | 82786 (117978) | 55467 (62762) | 0.54 |  | 3304 (10468) | 0 (0) | 0.32 |
| Total Z-SUM-4 score | 53278 (77876) | 33072 (41557) | 0.49 |  | 110 (364) | 0 (0) | 0.34 |
| Total Z-SUM-5 score | 33007 (50128) | 21341 (30310) | 0.54 |  | 0 (0) | 0 (0) | n.s. |
|  |  |  |  |  |  |  |  |
|  | Diabetes mellitus | | |  | Diabetes mellitus | | |
|  | No (n = 43) | Yes (n = 20) | P value |  | No (n = 19) | Yes (n = 3) | P value |
| Total Z-SUM-2 score | 96211 (112897) | 130690 (105439) | 0.25 |  | 13876 (41407) | 20873 (32894) | 0.78 |
| Total Z-SUM-3 score | 54025 (74318) | 69493 (64935) | 0.43 |  | 1833 (7992) | 501 (868) | 0.49 |
| Total Z-SUM-4 score | 32309 (49320) | 42793 (42859) | 0.42 |  | 64 (277) | 0 (0) | 0.33 |
| Total Z-SUM-5 score | 20290 (34221) | 28267 (30862) | 0.38 |  | 0 (0) | 0 (0) | n.s. |
|  |  |  |  |  |  |  |  |
|  | Dyslipidemia | | |  | Dyslipidemia | | |
|  | No (n = 14) | Yes (n = 49) | P value |  | No (n = 16) | Yes (n = 6) | P value |
| Total Z-SUM-2 score | 147846 (137203) | 95532 (100857) | 0.12 |  | 16037 (44985) | 11611 (23289) | 0.82 |
| Total Z-SUM-3 score | 82832 (88210) | 52108 (65180) | 0.16 |  | 2177 (8709) | 250 (614) | 0.39 |
| Total Z-SUM-4 score | 47698 (54973) | 32192 (44874) | 0.28 |  | 75 (302) | 0 (0) | 0.33 |
| Total Z-SUM-5 score | 27847 (33090) | 21387 (33373) | 0.52 |  | 0 (0) | 0 (0) | n.s. |
|  |  |  |  |  |  |  |  |
|  | Current smoking habit | | |  | Current smoking habit | | |
|  | No (n = 45) | Yes (n = 18) | P value |  | No (n = 21) | Yes (n = 1) | P value |
| Total Z-SUM-2 score | 108251 (120035) | 104422 (86984) | 0.90 |  | 14766 (40713) | 16166 (0) | 0.97 |
| Total Z-SUM-3 score | 58072 (74728) | 61094 (63952) | 0.88 |  | 1730 (7593) | 0 (0) | 0.83 |
| Total Z-SUM-4 score | 33371 (46964) | 41304 (48933) | 0.55 |  | 58 (264) | 0 (0) | 0.83 |
| Total Z-SUM-5 score | 19793 (30469) | 30397 (39000) | 0.25 |  | 0 (0) | 0 (0) | n.s. |
| *n.s.*, not significant; *Z-SUM*, sum of ^18^F-THK-5351 uptake intensity Z scores. | | | | | | | |
