# Supplementary material for: Investigation of reactive astrogliosis effect on post-stroke cognitive impairment
Source: J Neuroinflammation. 2020 Oct 17;17:308. doi: 10.1186/s12974-020-01985-0 (PMC7568828; doi:10.1186/s12974-020-01985-0)
Supplement: Supplementary file 4 — Additional file 4: Supplementary Table 3. Correlations of total Z-SUM scores with demographic, vascular and neurodegenerative features [file 12974_2020_1985_MOESM4_ESM.docx]

| **Supplementary Table 3.** Correlations of total Z-SUM scores with demographic, vascular and neurodegenerative features | | | | |
| --- | --- | --- | --- | --- |
|  | Total Z-SUM-2 | Total Z-SUM-3 | Total Z-SUM-4 | Total Z-SUM-5 |
| Age, y | 0.24 | 0.18 | 0.11 | 0.01 |
| Education, y | -0.11 | -0.14 | -0.14 | -0.14 |
| NIHSS | 0.23 | 0.24 | 0.20 | 0.14 |
| Stroke volume, % | 0.45*** | 0.55*** | 0.60*** | 0.58*** |
| PVL score | -0.17 | -0.21 | -0.25 | -0.26* |
| DWML score | 0.25* | 0.22 | 0.19 | 0.16 |
| Enlarged perivascular space | 0.04 | 0.05 | 0.04 | 0.04 |
| Lobar cerebral microbleeds | 0.05 | 0.01 | -0.05 | -0.09 |
| Deep cerebral microbleeds | -0.01 | -0.04 | -0.07 | -0.07 |
| Lacunes | -0.09 | -0.16 | -0.20 | -0.21 |
| MTA score | -0.04 | -0.07 | -0.10 | -0.13 |
| Cortical thickness, mm | -0.26* | -0.25 | -0.23 | -0.18 |
| Days between stroke onset and ^18^F-THK-5351 scanning | -0.04 | 0.02 | 0.09 | 0.11 |
| *DWML*, deep white matter leukoaraiosis; *MTA*, medial temporal atrophy; *NIHSS*, National Institutes of Health Stroke Scale; *PVL*, periventricular leukoaraiosis; *Z-SUM*, sum of ^18^F-THK-5351 uptake intensity Z scores. | | | | |
| * P < 0.05. ** P < 0.01. *** P < 0.001. | | | | |
