# Supplementary material for: Investigation of reactive astrogliosis effect on post-stroke cognitive impairment
Source: J Neuroinflammation. 2020 Oct 17;17:308. doi: 10.1186/s12974-020-01985-0 (PMC7568828; doi:10.1186/s12974-020-01985-0)
Supplement: Supplementary file 5 — Additional file 5: Supplementary Table 4. Associations of total Z-SUM scores of 18F-THK-5351 uptake intensity with cognitive function in model I [file 12974_2020_1985_MOESM5_ESM.docx]

| **Supplementary Table 4.** Associations of total Z-SUM scores of ^18^F-THK-5351 uptake intensity with cognitive function in model I^a^ | | | | | | | | | | | |
| --- | --- | --- | --- | --- | --- | --- | --- | --- | --- | --- | --- |
|  | Total Z-SUM-2 score | |  | Total Z-SUM-3 score | |  | Total Z-SUM-4 score | |  | Total Z-SUM-5 score | |
|  | β (SE), 10^-6^ | P value |  | β (SE), 10^-6^ | P value |  | β (SE), 10^-6^ | P value |  | β (SE), 10^-6^ | P value |
| MoCA | -6.8 (4.5) | 0.136 |  | -12.1 (6.8) | 0.082 |  | -18.9 (10.2) | 0.069 |  | -22.4 (14.6) | 0.131 |
| NPI | 9.5 (5.9) | 0.112 |  | 15.3 (9.3) | 0.105 |  | 23.0 (13.8) | 0.100 |  | 25.4 (19.0) | 0.186 |
| IADL | 1.7 (0.6) | 0.004 |  | 2.9 (0.9) | 0.002 |  | 4.1 (1.3) | 0.003 |  | 4.5 (2.0) | 0.024 |
| IQCODE^b^ | 1.2 (0.3) | 0.001 |  | 1.9 (0.5) | 0.001 |  | 2.5 (0.8) | 0.003 |  | 2.8 (1.1) | 0.015 |
| CDR-SOB | 2.5 (1.2) | 0.050 |  | 5.7 (1.9) | 0.004 |  | 8.4 (2.6) | 0.005 |  | 9.1 (3.9) | 0.025 |
| Composite cognitive *z* score |  |  |  |  |  |  |  |  |  |  |  |
| General cognitive function | -1.8(0.8) | 0.042 |  | -3.6 (1.3) | 0.008 |  | -5.7 (1.9) | 0.005 |  | -7.3 (2.8) | 0.011 |
| Memory function | -- | n.s. |  | -- | n.s. |  | -2.7 (2.9) | 0.356 |  | -3.7 (4.1) | 0.345 |
| Visuospatial function | -1.6 (0.9) | 0.076 |  | -2.6 (1.3) | 0.060 |  | -4.1 (2.0) | 0.046 |  | -5.6 (2.8) | 0.055 |
| Executive function | -2.9 (1.1) | 0.011 |  | -6.7 (1.8) | 0.000 |  | -10.5 (2.6) | 0.000 |  | -14.1 (3.8) | 0.001 |
| Language function | -1.5 (0.9) | 0.101 |  | -3.0 (1.4) | 0.035 |  | -5.0 (2.1) | 0.017 |  | -7.0 (2.9) | 0.019 |
| *CDR*, clinical dementia rating; *IADL*, instrumental activities of daily living; *IQCODE*, informant questionnaire on cognitive decline in the elderly; *MoCA*, Montreal cognitive assessment; *NPI*, neuropsychiatric inventory; *n.s.*, not significant; *SOB*, sum of boxes; *Z-SUM*, sum of ^18^F-THK-5351 uptake intensity Z scores. | | | | | | | | | | | |
| ^a^ Age and education as the confounding factors in the multiple linear regression model with forward stepwise variable selection. | | | | | | | | | | | |
| ^b^ Performed around 3 months after stroke. | | | | | | | | | | | |
