# Supplementary material for: Investigation of reactive astrogliosis effect on post-stroke cognitive impairment
Source: J Neuroinflammation. 2020 Oct 17;17:308. doi: 10.1186/s12974-020-01985-0 (PMC7568828; doi:10.1186/s12974-020-01985-0)
Supplement: Supplementary file 6 — Additional file 6: Supplementary Table 5. Associations of total Z-SUM scores of 18F-THK-5351 uptake intensity with cognitive function in model II [file 12974_2020_1985_MOESM6_ESM.docx]

| **Supplementary Table 5.** Associations of total Z-SUM scores of ^18^F-THK-5351 uptake intensity with cognitive function in model II^a^ | | | | | | | | | | | |
| --- | --- | --- | --- | --- | --- | --- | --- | --- | --- | --- | --- |
|  | Total Z-SUM-2 score | |  | Total Z-SUM-3 score | |  | Total Z-SUM-4 score | |  | Total Z-SUM-5 score | |
|  | β (SE), 10^-6^ | P value |  | β (SE), 10^-6^ | P value |  | β (SE), 10^-6^ | P value |  | β (SE), 10^-6^ | P value |
| MoCA | -- | n.s. |  | -- | n.s. |  | -9.9 (9.0) | 0.275 |  | -12.7 (12.7) | 0.322 |
| NPI | -- | n.s. |  | -- | n.s. |  | -- | n.s. |  | -- | n.s. |
| IADL | 1.3 (0.5) | 0.017 |  | 2.2 (0.8) | 0.008 |  | 3.3 (1.2) | 0.007 |  | 3.7 (1.7) | 0.035 |
| IQCODE^b^ | 0.8 (0.3) | 0.017 |  | 1.3 (0.5) | 0.013 |  | 1.6 (0.7) | 0.029 |  | 1.0 (1.2) | 0.443 |
| CDR-SOB | -- | n.s. |  | 1.9 (1.9) | 0.330 |  | 5.3 (2.3) | 0.026 |  | 6.6 (3.3) | 0.053 |
| Composite cognitive *z* score |  |  |  |  |  |  |  |  |  |  |  |
| General cognitive function | -0.9 (0.9) | 0.332 |  | -3.6 (1.3) | 0.008 |  | -5.7 (1.9) | 0.005 |  | -6.6 (2.7) | 0.017 |
| Memory function | -- | n.s. |  | -- | n.s. |  | -5.7 (2.8) | 0.044 |  | -6.1 (3.9) | 0.129 |
| Visuospatial function | -- | n.s. |  | -- | n.s. |  | -2.0 (2.5) | 0.432 |  | -2.8 (3.4) | 0.413 |
| Executive function | -1.5 (1.2) | 0.200 |  | -6.7 (1.8) | 0.000 |  | -10.5 (2.6) | 0.000 |  | -14.1 (3.8) | 0.001 |
| Language function | -- | n.s. |  | -1.8 (1.7) | 0.303 |  | -3.2 (2.6) | 0.231 |  | -3.9 (3.6) | 0.287 |
| *CDR*, clinical dementia rating; *IADL*, instrumental activities of daily living; *IQCODE*, informant questionnaire on cognitive decline in the elderly; *MoCA*, Montreal cognitive assessment; *NPI*, neuropsychiatric inventory; *n.s.*, not significant; *SOB*, sum of boxes; *Z-SUM*, sum of ^18^F-THK-5351 uptake intensity Z scores. | | | | | | | | | | | |
| ^a^ Age, education, National Institutes of Health Stroke Scale, stroke volume, periventricular leukoaraiosis and deep white matter leukoaraiosis as the confounding factors in the multiple linear regression model with forward stepwise variable selection. | | | | | | | | | | | |
| ^b^ Performed around 3 months after stroke. | | | | | | | | | | | |
