# Supplementary material for: Investigation of reactive astrogliosis effect on post-stroke cognitive impairment
Source: J Neuroinflammation. 2020 Oct 17;17:308. doi: 10.1186/s12974-020-01985-0 (PMC7568828; doi:10.1186/s12974-020-01985-0)
Supplement: Supplementary file 7 — Additional file 7: Supplementary Table 6. Associations of total Z-SUM scores of 18F-THK-5351 uptake intensity with cognitive function in model III [file 12974_2020_1985_MOESM7_ESM.docx]

| **Supplementary Table 6.** Associations of total Z-SUM scores of ^18^F-THK-5351 uptake intensity with cognitive function in model III^a^ | | | | | | | | | | | |
| --- | --- | --- | --- | --- | --- | --- | --- | --- | --- | --- | --- |
|  | Total Z-SUM-2 score | |  | Total Z-SUM-3 score | |  | Total Z-SUM-4 score | |  | Total Z-SUM-5 score | |
|  | β (SE), 10^-6^ | P value |  | β (SE), 10^-6^ | P value |  | β (SE), 10^-6^ | P value |  | β (SE), 10^-6^ | P value |
| MoCA | -- | n.s. |  | -- | n.s. |  | -- | n.s. |  | -- | n.s. |
| NPI | -- | n.s. |  | -- | n.s. |  | -- | n.s. |  | -- | n.s. |
| IADL | 1.3 (0.5) | 0.017 |  | 2.2 (0.8) | 0.008 |  | 3.3 (1.2) | 0.007 |  | 3.7 (1.7) | 0.035 |
| IQCODE^b^ | 0.8 (0.3) | 0.017 |  | 1.3 (0.5) | 0.013 |  | 1.6 (0.7) | 0.029 |  | -- | n.s. |
| CDR-SOB | -- | n.s. |  | 1.9 (1.9) | 0.328 |  | 5.3 (2.3) | 0.026 |  | 6.6 (3.3) | 0.053 |
| Composite cognitive *z* score |  |  |  |  |  |  |  |  |  |  |  |
| General cognitive function | -0.9 (0.9) | 0.332 |  | -3.6 (1.3) | 0.008 |  | -5.7 (1.9) | 0.005 |  | -6.6 (2.7) | 0.017 |
| Memory function | -- | n.s. |  | -- | n.s. |  | -4.4 (2.9) | 0.128 |  | -6.1 (4.0) | 0.134 |
| Visuospatial function | -- | n.s. |  | -- | n.s. |  | -- | n.s. |  | -- | n.s. |
| Executive function | -1.5 (1.2) | 0.200 |  | -6.7 (1.8) | 0.000 |  | -10.5 (2.6) | 0.000 |  | -14.1 (3.8) | 0.001 |
| Language function | -- | n.s. |  | -1.8 (1.7) | 0.303 |  | -3.2 (2.6) | 0.231 |  | -3.9 (3.6) | 0.287 |
| *CDR*, clinical dementia rating; *IADL*, instrumental activities of daily living; *IQCODE*, informant questionnaire on cognitive decline in the elderly; *MoCA*, Montreal cognitive assessment; *NPI*, neuropsychiatric inventory; *n.s.*, not significant; *SOB*, sum of boxes; *Z-SUM*, sum of ^18^F-THK-5351 uptake intensity Z scores. | | | | | | | | | | | |
| ^a^ Age, education, National Institutes of Health Stroke Scale, stroke volume, periventricular leukoaraiosis, deep white matter leukoaraiosis, medial temporal atrophy and cortical thickness as the confounding factors in the multiple linear regression model with forward stepwise variable selection. | | | | | | | | | | | |
| ^b^ Performed around 3 months after stroke. | | | | | | | | | | | |
