# Supplementary material for: Investigation of reactive astrogliosis effect on post-stroke cognitive impairment
Source: J Neuroinflammation. 2020 Oct 17;17:308. doi: 10.1186/s12974-020-01985-0 (PMC7568828; doi:10.1186/s12974-020-01985-0)
Supplement: Supplementary file 8 — Additional file 8: Supplementary Table 7. Demographic data, imaging findings and cognitive results between patients with left and right hemisphere stroke [file 12974_2020_1985_MOESM8_ESM.docx]

| **Supplementary Table 7.** Demographic data, imaging findings and cognitive results between patients with left and right hemisphere stroke^a^ | | | |
| --- | --- | --- | --- |
|  | Mean (SD) | | |
| Characteristics | Left (n = 30) | Right (n = 28) | P Value |
| Age, y | 64.3 (9.2) | 63.1 (8.3) | 0.61 |
| Education, y | 8.9 (4.2) | 9.8 (3.8) | 0.44 |
| Male, No. (%) | 20 (67) | 23 (82) | 0.18 |
| APOE ε4 carrier, No. (%) | 1 (3) | 5 (18) | 0.10^b^ |
| Common vascular risk factors |  |  |  |
| Hypertension, No. (%) | 26 (87) | 24 (86) | 1.00^b^ |
| Diabetes mellitus, No. (%) | 8 (27) | 12 (43) | 0.19 |
| Dyslipidemia, No. (%) | 21 (70) | 25 (89) | 0.06 |
| Gout, No. (%) | 7 (23) | 3 (11) | 0.30^b^ |
| PVL score | 0.5 (1.0) | 0.6 (0.8) | 0.77 |
| DWML score | 3.0 (1.2) | 2.9 (1.3) | 0.91 |
| Enlarged perivascular space, No. (%) | 10 (33) | 9 (32) | 0.92 |
| Lobar microbleed, No. (%) | 6 (20) | 4 (14) | 0.73^b^ |
| Deep microbleed, No. (%) | 3 (10) | 4 (14) | 0.70^b^ |
| Lacune, No. (%) | 4 (13) | 9 (32) | 0.08 |
| Stroke features |  |  |  |
| NIHSS | 2.0 (1.6) | 1.8 (2.1) | 0.77 |
| Ischemia stroke, No. (%) | 28 (93) | 25 (89) | 0.67^b^ |
| Stroke volume, % | 4.48E-6 (5.36E-6) | 5.04E-6 (6.01E-6) | 0.71 |
| MTA score | 1.1 (1.2) | 0.9 (1.1) | 0.57 |
| Cortical thickness, mm | 2.40 (0.11) | 2.42 (0.07) | 0.33 |
| Total Z-SUM score at different Z levels |  |  |  |
| Total Z-SUM-2, Z > 2 | 114331 (103394) | 116307 (122252) | 0.95 |
| Total Z-SUM-3, Z > 3 | 63159 (69143) | 64299 (76964) | 0.95 |
| Total Z-SUM-4, Z > 4 | 39550 (43451) | 37318 (47772) | 0.86 |
| Total Z-SUM-5, Z > 5 | 26493 (37069) | 22647 (30804) | 0.67 |
| MoCA | 21.3 (4.9) | 22.9 (4.9) | 0.23 |
| NPI | 3.4 (5.7) | 2.6 (3.9) | 0.54 |
| IADL | 1.4 (0.6) | 1.4 (0.6) | 0.98 |
| Depressive symptoms^c^ | 0.6 (1.6) | 0.7 (1.7) | 0.73 |
| Anxiety^d^ | 0.1 (0.6) | 0.1 (0.4) | 0.87 |
| IQCODE^e^ | 3.3 (0.3) | 3.3 (0.4) | 0.68 |
| CDR-SOB | 0.6 (1.0) | 0.7 (1.3) | 0.80 |
| Composite cognitive *z* score |  |  |  |
| General cognitive function | -0.97 (0.89) | -0.70 (0.79) | 0.23 |
| Memory function | -1.21 (1.11) | -0.98 (1.18) | 0.43 |
| Visuospatial function | -0.40 (0.85) | -0.15 (0.72) | 0.23 |
| Executive function | -0.84 (1.18) | -0.50 (0.93) | 0.24 |
| Language function | -1.15 (1.05) | -0.91 (1.05) | 0.38 |
| *APOE ε4*, apolipoprotein E ε4; *CDR*, clinical dementia rating; *DWML*, deep white matter leukoaraiosis; *IADL*, instrumental activities of daily living; *IQCODE*, informant questionnaire on cognitive decline in the elderly; *MoCA*, Montreal cognitive assessment; *MTA*, medial temporal atrophy; *NIHSS*, National Institutes of Health Stroke Scale; *NPI*, neuropsychiatric inventory; *PSCI,* post-stroke cognitive impairment; *PVL*, periventricular leukoaraiosis; *SOB*, sum of boxes; *Z-SUM*, sum of ^18^F-THK-5351 uptake intensity Z scores. | | | |
| ^a^ Unless otherwise indicated, data are expressed as mean (SD). | | | |
| ^b^ Analyzed by Fisher Exact test. | | | |
| ^c^ Evaluated by the NPI depression item 4. | | | |
| ^d^ Evaluated by the NPI anxiety item 5. | | | |
| ^e^ Performed around 3 months after stroke. | | | |
