# Supplementary material for: Investigation of reactive astrogliosis effect on post-stroke cognitive impairment
Source: J Neuroinflammation. 2020 Oct 17;17:308. doi: 10.1186/s12974-020-01985-0 (PMC7568828; doi:10.1186/s12974-020-01985-0)
Supplement: Supplementary file 9 — Additional file 9: Supplementary Table 8. Correlations of contralateral Z-SUM scores with cognitive performance in patients with left and right hemisphere stroke [file 12974_2020_1985_MOESM9_ESM.docx]

| **Supplementary Table 8.** Correlations of contralateral Z-SUM scores with cognitive performance in patients with left and right hemisphere stroke | | | | | | | | | |
| --- | --- | --- | --- | --- | --- | --- | --- | --- | --- |
|  |  |  |  |  |  |  |  |  |  |
| Patients with left hemisphere stroke (n = 30) | | | | | | | | | |
|  | MoCA | IADL | IQCODE^a^ | CDR-SOB | General cognitive function | Memory | Visuospatial function | Executive function | Language |
| Contralateral Z-SUM-2 | -0.02 | 0.16 | 0.26 | 0.15 | -0.18 | -0.2 | -0.19 | -0.10 | -0.11 |
| Contralateral Z-SUM-3 | 0.08 | 0.07 | 0.14 | 0.08 | -0.13 | -0.14 | -0.07 | -0.09 | -0.10 |
| Contralateral Z-SUM-4 | 0.16 | -0.07 | 0.03 | 0.00 | -0.04 | -0.10 | -0.02 | 0.01 | -0.02 |
| Contralateral Z-SUM-5 | 0.19 | -0.12 | 0.05 | -0.03 | 0.00 | -0.06 | 0.04 | 0.04 | 0.03 |
|  | | | | | | | | | |
| Patients with right hemisphere stroke (n = 28) | | | | | | | | | |
|  | MoCA | IADL | IQCODE^a^ | CDR-SOB | General cognitive function | Memory | Visuospatial function | Executive function | Language |
| Contralateral Z-SUM-2 | -0.08 | 0.10 | 0.24 | 0.20 | 0.07 | 0.12 | 0.01 | -0.02 | 0.04 |
| Contralateral Z-SUM-3 | -0.09 | 0.08 | 0.23 | 0.23 | 0.03 | 0.06 | 0.06 | -0.04 | 0.04 |
| Contralateral Z-SUM-4 | -0.06 | -0.02 | 0.15 | 0.14 | 0.06 | 0.02 | 0.06 | 0.06 | 0.04 |
| Contralateral Z-SUM-5 | -0.04 | -0.07 | 0.00 | 0.17 | 0.01 | -0.05 | -0.02 | 0.12 | -0.06 |
| *CDR*, clinical dementia rating; *IADL*, instrumental activities of daily living; *IQCODE*, informant questionnaire on cognitive decline in the elderly; *MoCA*, Montreal cognitive assessment; *NIHSS*, National Institutes of Health Stroke Scale; *NPI*, neuropsychiatric inventory; *SOB*, sum of boxes; *Z-SUM*, sum of ^18^F-THK-5351 uptake intensity Z scores. | | | | | | | | | |
| ^a^ Performed around 3 months after stroke. | | | | | | | | | |
