# Supplementary material for: Investigation of reactive astrogliosis effect on post-stroke cognitive impairment
Source: J Neuroinflammation. 2020 Oct 17;17:308. doi: 10.1186/s12974-020-01985-0 (PMC7568828; doi:10.1186/s12974-020-01985-0)

**Supplementary Figure 2.** Path analyses of stroke effects on cognition via total or ipsilateral Z-SUM-4 scores after age, education and anxiety adjustment. Stroke volume contributes significantly to cognitive function as well as total and ipsilateral Z-SUM-4 scores in patients with either left-hemispheric **(a, b**) or right-hemispheric (**c, d**) stroke. Further, ipsilateral Z-SUM-4 scores can partly mediate language influence from stroke volume in patients with left-hemispheric stroke (**b**). Likewise, total and ipsilateral Z-SUM-4 scores can partly mediate executive influence from stroke volume in patients with right-hemispheric stroke (**c, d**).


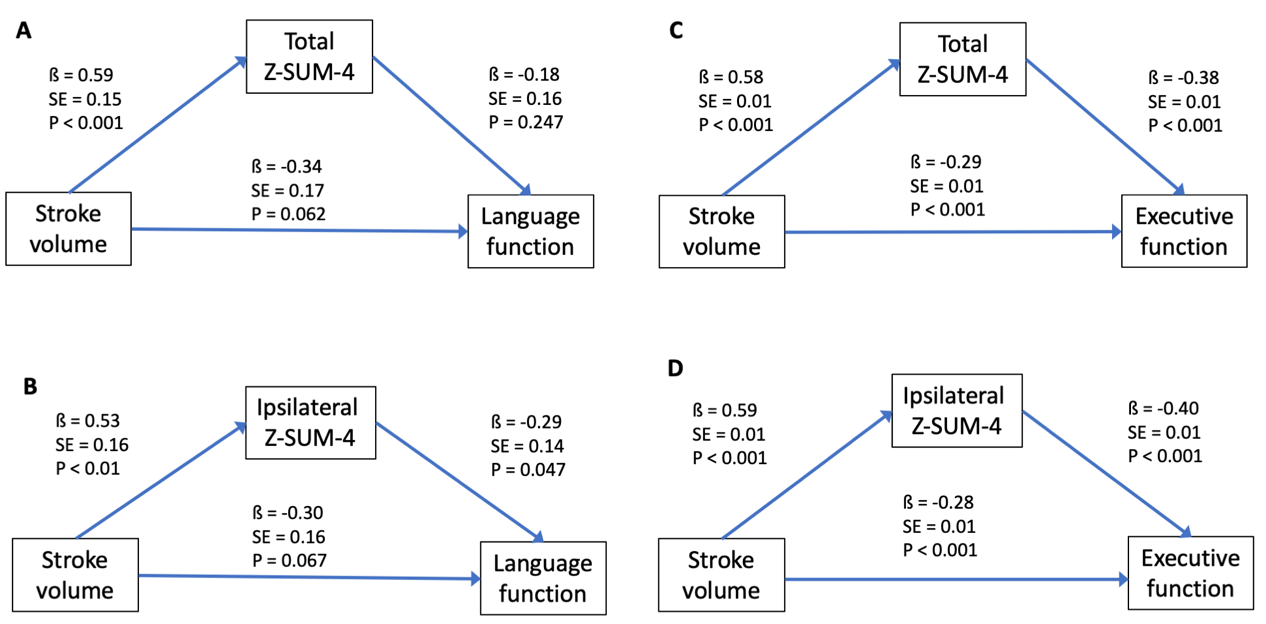

Supplement: Supplementary file 10 — Additional file 10: Supplementary Figure 2. Path analyses of stroke effects on cognition via total or ipsilateral Z-SUM-4 scores after age, education and anxiety adjustment. Stroke volume contributes significantly to cognitive function as well as total and ipsilateral Z-SUM-4 scores in patients with either left-hemispheric (a, b) or right-hemispheric (c, d) stroke. Further, ipsilateral Z-SUM-4 scores can partly mediate language influence from stroke volume in patients with left-hemispheric stroke (b). Likewise, total and ipsilateral Z-SUM-4 scores can partly mediate executive influence from stroke volume in patients with right-hemispheric stroke (c, d) [file 12974_2020_1985_MOESM10_ESM.docx]
