# Supplementary material for: Investigation of reactive astrogliosis effect on post-stroke cognitive impairment
Source: J Neuroinflammation. 2020 Oct 17;17:308. doi: 10.1186/s12974-020-01985-0 (PMC7568828; doi:10.1186/s12974-020-01985-0)
Supplement: Supplementary file 11 — Additional file 11: Supplementary Table 9. Effects of stroke volume and Z-SUM-4 score on cognitive function after adjustment for age, education and depressive symptoms (A~D) and anxiety (E~H) [file 12974_2020_1985_MOESM11_ESM.docx]

| **Supplementary Table 9.** Effects of stroke volume and Z-SUM-4 score on cognitive function after adjustment for age, education and depressive symptoms (A~D) and anxiety (E~H). | | | | | | | | |
| --- | --- | --- | --- | --- | --- | --- | --- | --- |
| Left hemisphere stroke | A. Language function | | |  | Right hemisphere stroke | B. Executive function | | |
|  | Total effect | Direct effect | Indirect effect |  |  | Total effect | Direct effect | Indirect effect |
| Stroke volume | -0.46 | -0.33 | -0.13 |  | Stroke volume | -0.25 | 0.04 | -0.29 |
| Total Z-SUM-4 score | -0.22 | -0.22 | -- |  | Total Z-SUM-4 score | -0.50 | -0.50 | -- |
| Goodness of fit: Chi-square = 1.6688, degree of freedom = 3, p < 0.6439, GFI = 0.9811, NFI = 0.9664 | | | |  | Goodness of fit: Chi-square = 3888, degree of freedom = 3, p < 0.0001, GFI = 0.9030, NFI = 0.8235 | | | |
|  |  |  |  |  |  |  |  |  |
| Left hemisphere stroke | C. Language function | | |  | Right hemisphere stroke | D. Executive function | | |
|  | Total effect | Direct effect | Indirect effect |  |  | Total effect | Direct effect | Indirect effect |
| Stroke volume | -0.47 | -0.30 | -0.17 |  | Stroke volume | -0.25 | 0.06 | -0.31 |
| Ipsilateral Z-SUM-4 score | -0.32 | -0.32 | -- |  | Ipsilateral Z-SUM-4 score | -0.52 | -0.52 | -- |
| Goodness of fit: Chi-square = 1.6567, degree of freedom = 3, p < 0.6466, GFI = 0.9812, NFI = 0.9667 | | | |  | Goodness of fit: Chi-square = 3742, degree of freedom = 3, p < 0.001, GFI = 0.9057, NFI = 0.8317 | | | |
|  |  |  |  |  |  |  |  |  |
| Left hemisphere stroke | E. Language function | | |  | Right hemisphere stroke | F. Executive function | | |
|  | Total effect | Direct effect | Indirect effect |  |  | Total effect | Direct effect | Indirect effect |
| Stroke volume | -0.44 | -0.33 | -0.11 |  | Stroke volume | -0.51 | -0.29 | -0.22 |
| Total Z-SUM-4 score | -0.18 | -0.18 | -- |  | Total Z-SUM-4 score | -0.38 | -0.38 | -- |
| Goodness of fit: Chi-square = 3.1063, degree of freedom = 3, p < 0.3755, GFI = 0.9662, NFI = 0.9441 | | | |  | Goodness of fit: Chi-square = 3718, degree of freedom = 3, p < 0.0001, GFI = 0.9062, NFI = 0.7550 | | | |
|  |  |  |  |  |  |  |  |  |
| Left hemisphere stroke | G. Language function | | |  | Right hemisphere stroke | H. Executive function | | |
|  | Total effect | Direct effect | Indirect effect |  |  | Total effect | Direct effect | Indirect effect |
| Stroke volume | -0.46 | -0.30 | -0.16 |  | Stroke volume | -0.51 | -0.27 | -0.24 |
| Ipsilateral Z-SUM-4 score | -0.29 | -0.29 | -- |  | Ipsilateral Z-SUM-4 score | -0.40 | -0.40 | -- |
| Goodness of fit: Chi-square = 2.5654, degree of freedom = 3, p < 0.4636, GFI = 0.9716, NFI = 0.9532 | | | |  | Goodness of fit: Chi-square = 3556, degree of freedom = 3, p < 0.0001, GFI = 0.9093, NFI = 0.7677 | | | |
