# Supplementary material for: Investigation of reactive astrogliosis effect on post-stroke cognitive impairment
Source: J Neuroinflammation. 2020 Oct 17;17:308. doi: 10.1186/s12974-020-01985-0 (PMC7568828; doi:10.1186/s12974-020-01985-0)
Supplement: Supplementary file 12 — Additional file 12: Supplementary Table 10. Comparisons of the contralateral Z-SUM scores of stroke patients with the average Z-SUM scores of bilateral hemispheres of healthy subjects [file 12974_2020_1985_MOESM12_ESM.docx]

| **Supplementary Table 10.** Comparisons of the contralateral Z-SUM scores of stroke patients with the average Z-SUM scores of bilateral hemispheres of healthy subjects | | | | | |
| --- | --- | --- | --- | --- | --- |
|  | Contralateral Z-SUM scores of stroke patients |  | Average Z-SUM scores of healthy subjects |  | P value |
| Z-SUM-2 | 31880 (42413) |  | 7415 (19866) |  | <0.001 |
| Z-SUM-3 | 10538 (20867) |  | 826 (3709) |  | <0.01 |
| Z-SUM-4 | 3586 (11505) |  | 27 (129) |  | 0.02 |
| Z-SUM-5 | 1547 (8325) |  | 0 (0) |  | 0.16 |
| Z-SUM, sum of 18F-THK-5351 uptake intensity Z scores. | | | | | |
