# Supplementary material for: Investigation of reactive astrogliosis effect on post-stroke cognitive impairment
Source: J Neuroinflammation. 2020 Oct 17;17:308. doi: 10.1186/s12974-020-01985-0 (PMC7568828; doi:10.1186/s12974-020-01985-0)
Supplement: Supplementary file 13 — Additional file 13: Supplementary Table 11. The correlations between total Z-SUM scores and cognitive function in ischemic stroke patients [file 12974_2020_1985_MOESM13_ESM.docx]

| **Supplementary Table 11.** The correlations between total Z-SUM scores and cognitive function in ischemic stroke patients | | | | |
| --- | --- | --- | --- | --- |
|  | Total Z-SUM-2 | Total Z-SUM-3 | Total Z-SUM-4 | Total Z-SUM-5 |
| MoCA | -0.30* | -0.32*† | -0.29* | -0.22 |
| NPI | 0.25 | 0.23 | 0.21 | 0.17 |
| Depressive symptoms^a^ | 0.08 | 0.06 | 0.04 | 0.01 |
| Anxiety^b^ | -0.09 | -0.13 | -0.14 | -0.14 |
| IADL | 0.41**† | 0.43**† | 0.40**† | 0.32*† |
| IQCODE^c^ | 0.43**† | 0.44**† | 0.40**† | 0.31*† |
| CDR-SOB | 0.39**† | 0.42**† | 0.39**† | 0.29*† |
| Composite cognitive *z* score |  |  |  |  |
| General cognitive function | -0.31*† | -0.39**† | -0.39**† | -0.36**† |
| Memory function | -0.16 | -0.20 | -0.20 | -0.17 |
| Visuospatial function | -0.35**† | -0.33*† | -0.29*† | -0.24 |
| Executive function | -0.35**† | -0.44**† | -0.46**† | -0.43**† |
| Language function | -0.25† | -0.31*† | -0.33*† | -0.33*† |
| *CDR*, clinical dementia rating; *IADL*, instrumental activities of daily living; *IQCODE*, informant questionnaire on cognitive decline in the elderly; *MoCA*, Montreal cognitive assessment; *NPI*, neuropsychiatric inventory; *SOB*, sum of boxes; *Z-SUM*, sum of ^18^F-THK-5351 uptake intensity Z scores. | | | | |
| ^a^ Evaluated by the NPI depression item 4. | | | | |
| ^b^ Evaluated by the NPI anxiety item 5. | | | | |
| ^c^ Performed around 3 months after stroke. | | | | |
| * P < 0.05. ** P < 0.01. *** P < 0.001. † P < 0.05 after adjustment for age and education. | | | | |
