# Supplementary material for: Investigation of reactive astrogliosis effect on post-stroke cognitive impairment
Source: J Neuroinflammation. 2020 Oct 17;17:308. doi: 10.1186/s12974-020-01985-0 (PMC7568828; doi:10.1186/s12974-020-01985-0)
Supplement: Supplementary file 14 — Additional file 14: Supplementary Table 12. Clinical characteristics of patients with hemorrhagic and ischemic stroke [file 12974_2020_1985_MOESM14_ESM.docx]

| **Supplementary Table 12.** Clinical characteristics of patients with hemorrhagic and ischemic stroke^a^ | | | |
| --- | --- | --- | --- |
|  | Mean (SD) |  |  |
| Characteristics | Hemorrhagic stroke  (n = 5) | Ischemic stroke  (n = 58) | P value |
| Age, y | 58 (5.4) | 64.6 (8.9) | 0.11 |
| Education, y | 10.2 (2.7) | 9.2 (4.1) | 0.59 |
| Male, No. (%) | 4 (80) | 43 (74) | 1.00^b^ |
| APOE ε4 carrier, No. (%) | 0 (0) | 7 (12) | 1.00^b^ |
| Days between stroke onset and cognition evaluation | 108 (29) | 102 (26) | 0.61 |
| Days between stroke onset and ^18^F-THK-5351 scanning | 89 (17) | 98 (20) | 0.32 |
| Days between stroke onset and ^18^F-florbetapir scanning | 112 (38) | 100 (30) | 0.38 |
| Common vascular risk factors |  |  |  |
| Hypertension | 5 (100) | 50 (86) | 1.00^b^ |
| Diabetes mellitus | 0 (0) | 20 (34) | 0.17^b^ |
| Dyslipidemia | 4 (80) | 45 (78) | 1.00^b^ |
| Gout, No. (%) | 1 (20) | 9 (16) | 1.00^b^ |
| PVL score | 0.8 (0.8) | 0.5 (0.9) | 0.49 |
| DWML score | 3.4 (1.7) | 2.8 (1.2) | 0.29 |
| NIHSS | 2.8 (1.8) | 1.8 (1.8) | 0.22 |
| Stroke volume, % | 4.14E-6 (1.67E-6) | 4.53E-6 (5.73E-6) | 0.72 |
| MTA score | 0.4 (0.9) | 1 (1.1) | 0.23 |
| Cortical thickness, mm | 2.43 (0.05) | 2.41 (0.09) | 0.73 |
| Total Z-SUM scores at different Z levels | |  |  |
| Total Z-SUM-2, Z > 2 | 167040 (143191) | 101995 (107711) | 0.21 |
| Total Z-SUM-3, Z > 3 | 80355 (74055) | 57089 (71442) | 0.49 |
| Total Z-SUM-4, Z > 4 | 29740 (23467) | 36146 (48877) | 0.77 |
| Total Z-SUM-5, Z > 5 | 11992 (10216) | 23756 (34309) | 0.09 |
| *APOE ε4*, apolipoprotein E ε4; *DWML*, deep white matter leukoaraiosis; *MTA*, medial temporal atrophy; *NIHSS*, National Institutes of Health Stroke Scale; *PSCI*, post-stroke cognitive impairment; *PVL*, periventricular leukoaraiosis; *Z-SUM*, sum of ^18^F-THK-5351 uptake intensity Z scores. | | | |
| ^a^ Unless otherwise indicated, data are expressed as mean (SD). | | | |
| ^b^ Analyzed by Fisher's exact test. | | | |
